# Phytochemistry, Biological, and Pharmacological Properties of *Abies alba* Mill.

**DOI:** 10.3390/plants12152860

**Published:** 2023-08-03

**Authors:** Robert Ancuceanu, Marilena Viorica Hovaneț, Anca Miron, Adriana Iuliana Anghel, Mihaela Dinu

**Affiliations:** 1 Faculty of Pharmacy, Carol Davila University of Medicine and Pharmacy, 020956 Bucharest, Romania; adriana.anghel@umfcd.ro (A.I.A.); mihaela.dinu@umfcd.ro (M.D.); 2 Faculty of Pharmacy, Grigore T. Popa University of Medicine and Pharmacy, 700115 Iasi, Romania; anca.miron@umfiasi.ro

**Keywords:** *Abies alba* Mill., Pinaceae, essential oils, extracts, resin, monoterpenes, sesquiterpenes, lignans, polyphenols, antimicrobial, antioxidant

## Abstract

*Abies alba* Mill. (Pinaceae), silver fir, is a widespread gymnosperm species in Europe, important for its ecological, economic, social, and cultural significance, as well as for its use for food and bioremediation purposes. The various parts of the plant (leaves, branches, cones, wood, bark) are also of pharmaceutical interest due to their composition of active compounds. In the last three decades, an impressive amount of research has been dedicated to this species. The variability of the chemical composition of essential oils (whether they come from leaves, oleoresin from branches, or other parts of the plant) is impressive, even in the case of specimens collected from the same geographical area. For essential oils prepared from needles or twigs and branches, limonene, β-pinene, α-pinene, camphene, β-phellandrene, and bornyl acetate are the leading compounds, although their wide variations seem to correspond to multiple chemotypes. Both bark and wood are rich in lignans and phenolic compounds. Matairesinol is apparently the dominant lignan in bark, and secoisolariciresinol and lariciresinol are the dominant ones in wood samples. Pharmacological studies with promising results have evaluated the antioxidant effect (mainly due to essential oils), but also the antimicrobial, antitumor, probiotic, antidiabetic, anti-steatosis, and anti-psoriatic activities.

## 1. Introduction

*Abies* is a genus of plants first described by Philipp Miller (1691–1771) in 1754. The second-largest genus of the family Pinaceae, it is considered more complex in comparison with other genera in this family, and all its taxa are natively distributed throughout the Northern Hemisphere [1,2]. It is speculated that the name Abies is derived from the Latin word *abeo*, meaning to depart, and interpreted as an allusion to its great height. The genus *Abies* is estimated to be 52 species with accepted status, to which 58 other species, whose status has not yet been ascertained, must be added [3].

*Abies alba* Mill. (Pinaceae), silver fir, is among the most widespread tree species in Europe (particularly Central and Eastern Europe, with marginal groups in the southern regions of Europe—Balkans and Pyrenees). Throughout Europe, it is important for its large environmental, economic, social, and cultural significance [4,5]. In Central Europe, silver fir is reported to be the most common tree species, whereas, in Switzerland, it represents about one-seventh of the whole forest growing stock [6,7]. 

**Figure 1:**
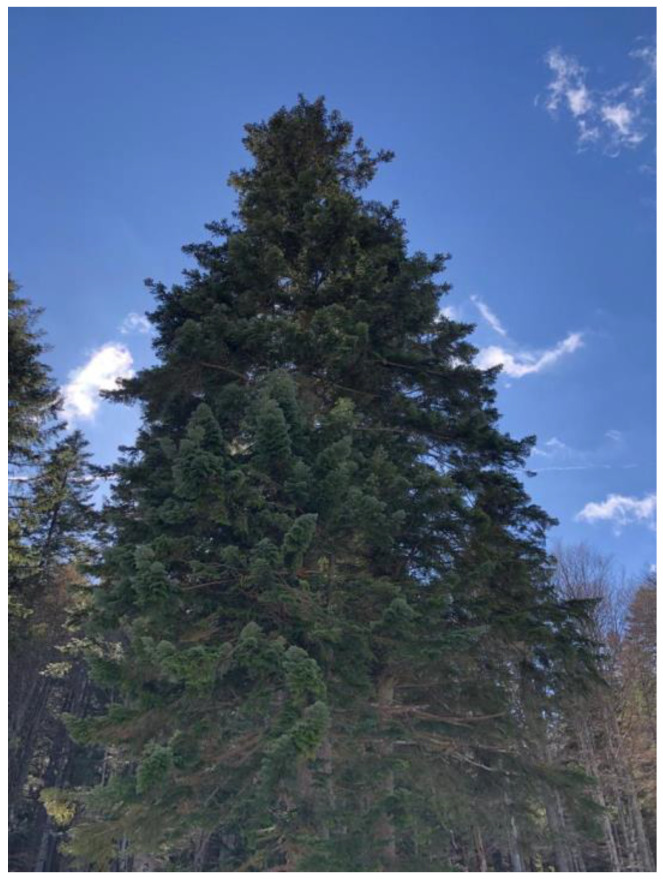
A silver fir (*Abies alba* Mill.) tree at the edge of a Romanian forest (Bușteni).

**Figure 2:**
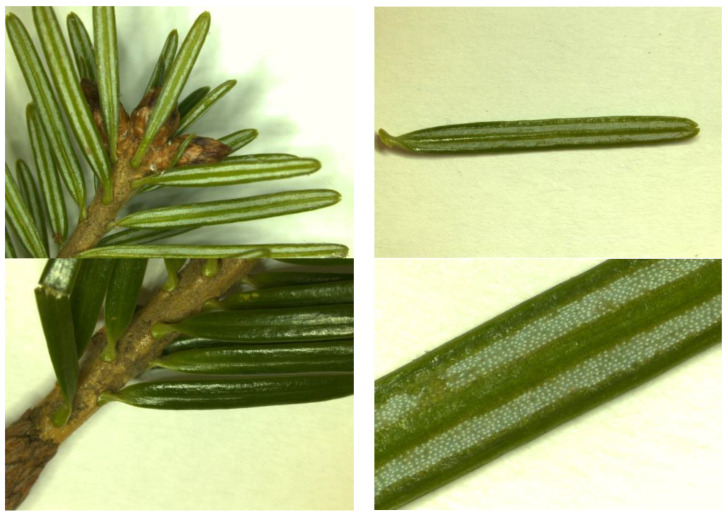
Adaxial and abaxial leaves of *A. alba* Mill inserted on a branch. The last image shows the stomata forming silverish bands on the abaxial side, examined with a Leica DMS 1000 digital microscope.

Young specimens grow slowly in the beginning, but the growth accelerates as the tree approaches maturity. Similar to most gymnosperms, it is monoecious, and it has a straight, monopodial, columnar stem, whereas its trunk bark is silver–greyish, smoother in young trees, and darker and cracked, with pinkish fractures in the older trees (Figure 1) [8,9,10]. *A. alba* is among the tallest species of its genus, reaching heights of up to 60 m [8]. Its leaves (“needles”) persist on the tree for 6–8 years and have a dark green color, while on the abaxial side, they show two silverish, longitudinal bands consisting of six–eight lines of stomata (the upper side of the needle, shiny and dark green, is devoid of stomata or only has a few near the tip) (Figure 2) [8,10]. The terminal and subterminal buds can have very high contents of resin [9]. Male cones tend to occur bellow female cones on the tree and are (purplish) reddish-to-yellowish, solitary, pendulous, similar to aments, having peltate microsporophylls, disposed in a spiral, and having two microsporangia (pollen sacs) [9]. Female cones tend to occur in the upper areas of the crown, are disposed axillary, solitary, and around the time of pollination, they are disposed erectly, being 3–5 cm long and greenish (with a purple touch when occurring late in the spring) [10,11]. When mature, female cones reach 8–20 cm in length and 3–5 cm in width. They are abundantly resinous and disintegrate as a result of the helically attached bracts, seed scales, and seeds falling from the long, rough, woody rachis. The latter remains persistent on the branches [9,10,11]. Seeds are 7–13 mm long, triangular, shiny, with reddish–yellowish wings, and 10–14 mm long [11]. Whereas bark, leaves, and cones contain resin canals, wood is devoid of such canals, except when traumatized [9]. From the perspective of its red-list status, *Abies alba* Mill. is a species of the least concern [12]. 

In the 19th century, its use for food purposes was first proposed in the form of silver fir beer [13]. Recently, an extract obtained from its needles was proposed to be used for whole wheat bread enrichment in a proportion of up to 35% (the threshold of taste perception), its addition increasing the antioxidant properties of the bread and the technological qualities of the dough and bread [14]. Its bark was proposed as a good sorbent for the removal of copper from effluents with a reduced copper content [15].

This narrative review is based on a comprehensive search in Pubmed, Web of Science, Scopus, and Google Scholar, using “Abies and alba”, “silver and fir”, or “silverfir” as keywords. References of the relevance of returned results were also used to identify potentially additional references, although, for a small number of cases, we were not able to retrieve the full text of papers older than from 1990. Publications in the English, French, or German languages were retained for analysis and synthesis in this review.

## 2. Parts Used and Extraction Methods

To date, silver fir needles have been particularly explored (in a large body of studies) for their essential oil contents. Instead, bark and wood have been investigated, particularly in the last two decades, for their lignan and phenolic contents. Whereas the nature of chemical compounds in each plant part will be discussed in the phytochemistry section of this paper, we here discuss some general aspects of different parts used for extraction purposes, extraction yields, and variables influencing them, and we mention several commercial extracts that have attracted interest in publications.

Although needles have been less explored to obtain extracts with various solvents, recently, the use of water to obtain cheap and nature-friendly extracts through hydrodynamic cavitation has been advanced [16], but their bioactivity has not as yet been reported. Extractives obtained from needles with an ethanol–toluene mixture were particularly rich in substances (higher yields), especially terpenes, compared with bark and cones [17]. 

Because silver fir is mostly used for its wood and, to some extent, for its resin and essential oils, its bark is rather a residue or byproduct that could be used for pharmaceutical purposes [4]. From an economic perspective, after wood, bark is considered the second-most useful product of forestry, and it may consist of 10–15% of the stem (in terms of volume) [18]. Not only is bark economically a by-product worth exploiting, but based on data from other Gymnosperm genera, it is estimated that bark is much richer in extractible chemical compounds than stem wood, containing up to six times higher amounts than the latter [18]. The chemical makeup of bark is affected by variations not only from tree to tree but even within the same individual, as with age, its tissues change their structure and composition [18]. A wide variation from tree to tree has been claimed for the monoterpene compounds from the cortical oleoresin [19]. In general, the oleoresin composition of conifers has been regarded as an expression of genotypic information because it is less sensitive to environmental influences. Therefore, it is considered particularly useful for chemotaxonomic purposes in the case of gymnosperms, as it allows the characterization of trees of different origins or clones within the same species [19]. The usefulness of resin produced by various plant tissues as a (chemo)taxonomic tool is based on the fact that it seems to be under the control of a relatively small number of genes and is little influenced by environmental variables [19]. 

An average content of 16.7% (*w*/*w*, d.w.) of water-extractable substances was reported in the bark of the stem and branches [18]. The mean content of water extractives did not differ significantly between the inner and outer layers of the bark (*p* = 0.62, ANOVA) [18], although it has been stated that (for other species) “the inner bark typically contains more extractives than the mature, outer bark” [20]. *n*-Hexane “extracts” (actually fractions of the hydro-ethanolic extract, i.e., lipophilic compounds) tended to increase significantly with height [20]. The total extraction yield from bark using a hydro-ethanolic mixture (50:50 *v*/*v*) tended to escalate from the stem base toward the crown, where it reaches a relatively stable plateau, being little influenced by the branches [20]. 

Wood is another byproduct of several industries that could be explored for the extraction of various chemical compounds, particularly lignans and phenolics. Compared with other *Abies* species (*A. sibirica* Ledeb, *A. lasiocarpa* (Hook.) Nutt., and *A. balsamea* (L.) Mill.), the extraction yields for *A. alba* are among the lowest for sapwood (0.70%, d.w.), among the highest for heartwood (2.1%, d.w.), and at the average levels for living knots (13%) and dead knots (15%) [21]. 

Knots are the bases of side branches or dormant buds inside the tree stem. Knotwood provides the highest extraction yield with both lipophilic (hexane) and hydrophilic (acetone–water) solvents (20.1 mg/g and 210.4 mg/g, respectively), whereas sapwood provides the lowest yield (4.7 mg/g and 14.8 mg/g, respectively) [22]. The extraction yield of heartwood was only slightly superior to the one of sapwood, whereas the extraction yield of living knots and branch wood was similar and somewhat inferior to the one of dead knots but definitely superior to sapwood and heartwood [22]. Extraction yields for both acetone and hexane as solvents tended to decrease for knot wood from the base of the crown toward the top [23]. Yields of hexane extraction from knot wood varied between 0.13% and 6.9%, depending on the position and schedule of thinning used. Young knots (from the stem tops) tended to have the lowest yields [24]. A similar relationship was recorded for the extraction with pure ethanol; knots sampled below the crown base provided even higher yields, but only in the case of un-thinned trees (whereas thinned trees generated weak yields) [23]. Acetone extracts obtained from knot wood varied considerably, between 1.02% and 34.1%, depending on the position and schedule of thinning. Young knots (from the stem top) had the lowest yields, whereas those close to the living crown base tended to have the highest yields. Loose zones of knots were richer in extractives than tight zones [24]. 

For branches, it was reported that optimal extraction might be obtained at a temperature of 100 °C, using a sample-to-water ratio of 1:10 and a relatively long extraction time (90 min). The extractable constituents of branches dropped by about 40% as the portion sampled was farther away from the trunk [13]. 

Cones have been reported to be very poor in the phenols and flavonoids [17], and this might be partially responsible for the lower interest in the phytochemical exploration of this part of the plant. 

Belinal^®^ is an industrially manufactured polyphenolic extract derived from *A. alba* branches (i.e., mostly wood), with in vitro antioxidant effects similar to those of epigallocatechin gallate and superior to those of resveratrol, ascorbic acid, or butylated hydroxytoluene [25]. It is actually the ethyl acetate-soluble fraction of an extract prepared with water at 70 °C for 2 h, and at least two scientific papers have been published on this extract [26]. 

Abigenol^®^ is another extract obtained with water in a similar manner to Belinal^®^, but from bark [25], at 70 °C. It is actually an ethyl-acetate soluble fraction of the aqueous extract thus obtained, suspended in polyethylene glycol 400, which is the reason why it has a liquid, viscous consistency [4]. Its polyphenolic profile is similar to that of Belinal^®^, and its antioxidant activity was claimed to be higher than that of Pycnogenol (a well-known commercial extract obtained from the bark of *Pinus maritima* Lam., syn. *Pinus pinaster* Aiton) [25]. 

A silver fir trunk extract (SFTE) has been prepared using water as an extraction solvent (DER 100:1) and standardized in protocatechuic acid (7.7 g/L) and *p*-coumaric acid (3.7 g/L). It is also the ethyl acetate-soluble fraction of an aqueous extract suspended in polyethylene glycol 400, and thus, it has a viscous, liquid consistency [27]. This is very similar to Belinal^®^ and Abigenol^®^, being obtained in the same manner; the only difference is that for Abigenol^®^; the plant part extracted is the bark [4]. 

## 3. Phytochemistry

*A. alba* Mill. has aroused interest in the field of phytochemistry in at least three major directions: essential oils contained in multiple parts of the plant; polyphenols (particularly in the bark); and lignans (especially in the bark and wood). 

### 3.1. Terpenoids and Other Components of Essential Oils

Most parts of the *A. alba* tree contain essential oils with similar but not identical compositions and production yields, the major compounds being monoterpene hydrocarbons, with smaller amounts of sesquiterpene hydrocarbons and only minor presences of oxygenated compounds (mostly monoterpenes, very few sesquiterpenes). Moreover, as it will be seen, there are wide inter- and intra-individual variations reported, with many composition patterns corresponding to an important number of chemotypes that have not yet been classified in a standardized manner. The first phytochemical studies regarding essential oils from *A. alba* Mill. seem to have been performed in the 1970s in Poland, attributed to J. Jończyk [28,29]. The essential oil obtained by steam distillation of fresh needles (fir needle oil) is known under the Latin name of *Abietis albae aetheroleum*, whereas the one prepared from cones is known as *Oleum templini*. From its resin, turpentine, known as Strasburg or Alsace turpentine, is obtained, reportedly containing essential oil, resin acids, bitter compounds, and succinic acid [30]. Other parts of the plant also contain essential oils; therefore, they will be discussed separately below for each source and, as the case may be, the method of preparation.

#### 3.1.1. Essential Oil from Leaves

**Figure 3:**
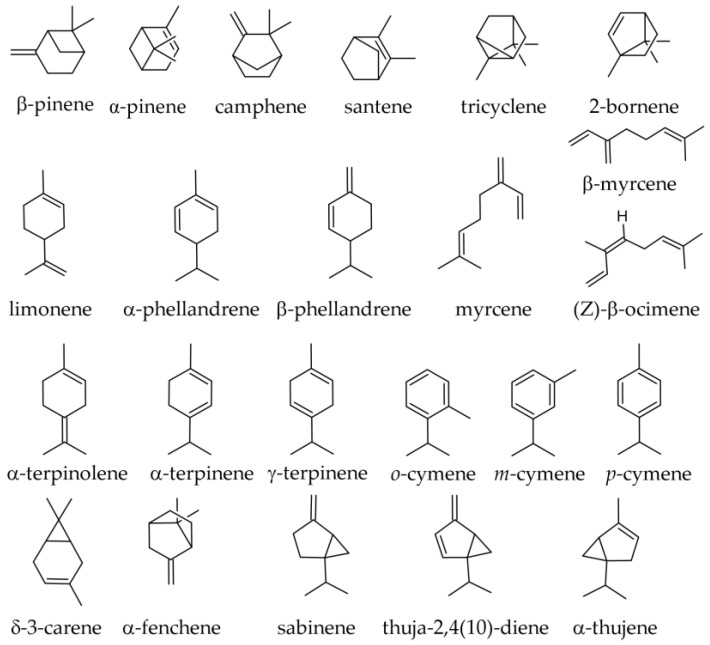
Monoterpene hydrocarbons identified in essential oils of various parts of *Abies alba* Mill.

The needles are relatively rich in monoterpenes, and some of these monoterpenes are emitted into the air. A study intended to estimate the monoterpene emissions by a silver fir forest in France reported that the main monoterpene emissions consisted of limonene (over 50% of all emissions), α-pinene, and camphene. The species is considered a weak monoterpene emitter (about 1 μg g^−1^ h^−1^, on a dry basis), its levels being similar to those of other conifers [31]. In samples collected from France (Collet d’Allevard forest), the following monoterpene hydrocarbons were reported as emitted by the foliage of the species (using an effluvial headspace sampler; it is to be assumed that the terpenes emitted reflect a good extent the terpenes contained in the needles) (Figure 3): α-pinene (52%); limonene and +β-phellandrene (not separated in the GC analysis) (21.1%); β-pinene (12.2%); myrcene (1.9%); camphene (0.9%); and in traces, tricyclene and sabinene [32]. 

Essential oils obtained from leaves have various applications in the cosmetic industry, particularly as an ingredient in air fresheners, fragrances/perfumes, and home products [33]. It has been appreciated that among the essential oils derived from the main European conifer needles, the one prepared from *A. alba* leaves “possesses perhaps the most pleasant odor”. Because of this property, this oil is widely used in Europe in a variety of products such as air fresheners, deodorants, bath preparations, inhalant products intended for the treatment of coughs and colds, as well as other pharmaceutical preparations such as ointments [34]. 

The essential oil obtained by hydrodistillation from needles of *A. alba* collected in Albania had a pleasant smell, and its color ranged from pale yellow to colorless. Yields varied between 0.36 and 0.88 mL/g of fresh tissue for needles collected during the winter (November–December) and between 0.13 and 0.40 for needles collected during the summer (May–June). In agreement with previous research, this reveals a decline in the production of needle oleoresin from fall to spring and a higher yield for winter samples than for summer samples [19].

All samples analyzed from different European countries reported limonene (34–55%—Germany, Austria, France, Greece) or β-pinene (20–33%—Serbia, Montenegro) as the leading compounds [1]. Other major compounds were also monoterpenes: camphene (15–17%); α-pinene (11–17%); and bornyl acetate (9–14%) [1]. In a sample prepared from leaves and twigs of Korean specimens, bornyl acetate was the dominant compound (30.31%), followed by camphene (19.81%), 3-carene (13.85%), and tricyclene [35]. The essential oil from *A. alba* seems to be poorer in α-pinene (31.2 ± 5.8% vs. 18.0 ± 3.6%) than *A. cephalonica* Loudon, whereas the contents in β-pinene do not differ significantly; instead, the essential oil from *A. alba* was richer in limonene + β-phellandrene (21.7 ± 11.1% vs. 10.4 ± 4.8%) [2]. The contents of both β-pinene and α-pinene seem to be lower in the essential oil of *A. alba* needles compared with that of *A. x borisii-regis* Mattf. needles (30.6 ± 11.0 vs. 38.0 ± 10.8% for β-pinene; 18.0 ± 3.6 vs. 24.2 ± 4.9% for α-pinene); instead, the limonene + β-phellandrene contents seem to be higher in *A. alba* than *A. x borisii-regis* (21.7 ± 11.1 vs. 12.9 ± 5.3) [2]. 

A wide variation from tree to tree has been claimed for the monoterpene compounds in leaf essential oils. In a study performed in Albania, three populations had a similar profile, with the leading compounds in the following order: β-pinene > camphene > α-pinene > limonene; a fourth population had a different chemical profile of the essential oil, with the leading compounds in the following order: β-pinene > α-pinene > limonene > camphene [19]. In samples from Montenegro, the following variation of the leading compounds was reported: β-pinene > α-pinene > camphene > bornyl acetate > limonene [34]. Another study based on samples from the Southern Carpathians and the Balkan Peninsula also reported what appears to be the same pattern: β-pinene > camphene > α-pinene > limonene [2], whereas a study based on samples from the South Balkans (Greece and Serbia) found a slightly differed pattern: β-pinene > camphene > limonene > α-pinene, the difference between limonene and α-pinene being so small to be virtually in equal proportion; however, in this sample, there was a high level of α-fenchyl acetate (14.2%), higher than the limonene or α-pinene contents [36]. A sample from Italy, as well as a commercial sample apparently from Germany, reported the following order of the leading compounds: limonene > α-pinene > camphene > β-pinene [37,38]. In samples from the Botanical Garden of the University of Würzburg, the leading compounds varied as follows: limonene > camphene > α-pinene > santene (the β-pinene level was very low, 0.5% only) [38]. 

β-Pinene had higher levels in Albanian samples than in samples from Southeastern Europe and Calabria; instead, α-pinene and camphene were reported in lower amounts in Albanian than in Eastern and Southeastern European samples. β-Phellandrene was reported with a mean concentration of 5.4% by Wolf (1994) (but a high variation, from 0% to 54%), whereas, in Albanian samples, it was completely absent [19]. In the samples from the Southern Carpathians and the Balkan Peninsula, β-phellandrene was reported together with limonene at 21.7% ± 11.1 [2], but it was apparently absent in a sample originating from Italy [37]. 

In the leaf essential oils derived from samples collected during the summer from several countries in Southern Carpathians and the Balkan Peninsula (Romania, Serbia, North Macedonia, and Bulgaria) by J.S. Nikolic et al. (2021), the leading compounds were largely monoterpene hydrocarbons (95.8% ± 2.4%); oxygenated monoterpenes represented 2.6 ± 1.8%, sesquiterpene hydrocarbons 1.5 ± 1.4%, whereas oxygenated sesquiterpenes were only detected in traces [2]. Instead, in essential oil samples from Serbia only (also from needles collected during the summer), different proportions for the terpenic compounds were reported by Z.S. Mitic et al. (2022): 66.4% monoterpene hydrocarbons; 12.1% oxygenated monoterpenes; 18.0% sesquiterpene hydrocarbons; and 1% oxygenated sesquiterpenes [39]. 

In essential oils obtained from the samples collected in Albania, the authors identified over 60 compounds, corresponding to about 92–96% of the oleoresin. In the essential oil obtained from Albanian samples, monoterpene hydrocarbons represented 70.3–77.2% of the samples prepared from needles collected in winter but only 40.3–47.9% of the samples prepared from needles collected in summer [19]. In the samples from the Southern Carpathians and the Balkan Peninsula, although they were also collected in summer–early autumn, monoterpene hydrocarbons amounted to 95.8 ± 2.4% [2]. In samples collected in the South Balkans (Greece and Serbia) around the end of August, monoterpene hydrocarbons represented 84.1% [36]. The same was the case for the samples collected in France (Collet d’Allevard forest) during the summer (mid-June): the authors reported exclusively on the monoterpenes [32]. Conversely, whereas sesquiterpene hydrocarbons represented 2.8–5.1% of the Albanian winter samples, their proportion covered 23.0–32.6% of the summer samples [19]. However, in the samples from the Southern Carpathians and the Balkan Peninsula, sesquiterpene hydrocarbons represented only 1.5 ± 1.4% [2]. Oxygenated monoterpenes tended to vary less, 10.1–22.05% for the winter samples and 11.3–21.4% for the summer samples; however, for individual compounds, as discussed below, there still may be considerable differences between the winter and summer samples [19]. 

The monoterpene hydrocarbons reported in winter and summer samples from Albania are shown in Table 1. Most monoterpene hydrocarbons tend to be less represented in the summer samples, α-terpinolene being the only one whose proportion increased in the summer samples.

Oxygenated monoterpenes reported in Albanian winter and summer samples are summarized in Table 2. Compared with winter samples, in summer samples, oxygenated monoterpenes show both increases and decreases. Bornyl acetate levels tended to correlate positively with camphene, whereas both bornyl acetate and camphene correlated negatively with borneol and α-terpineol contents [19].

In samples of *A. alba* needles collected in the Southern Carpathians and the Balkan Peninsula/South Balkans during the summer and early autumn, the following monoterpene hydrocarbons were reported: β-pinene (19.8–30.6%); limonene + β-phellandrene (21.7%); limonene (11.0%); camphene (10.9–19.2%); α-pinene (10.9–18.0%); α-fenchyl acetate (0.0–14.2%); tricyclene (1.7–3.9%); α-thujene (traces—2.8%); myrcene (0.9–1.3%); santene (0.5–0.8%); terpinolene (0.4–0.9%); α-phellandrene (0.1–0.9%); α-terpinene (traces—0.2%); γ-terpinene (traces—0.2%); and absent or in traces *o*-cymene and sabinene [2,36,39]. In samples from Albania, the limonene content was estimated at 10.7% and β-phellandrene at 6.3% [36,39]. 

**Figure 4:**
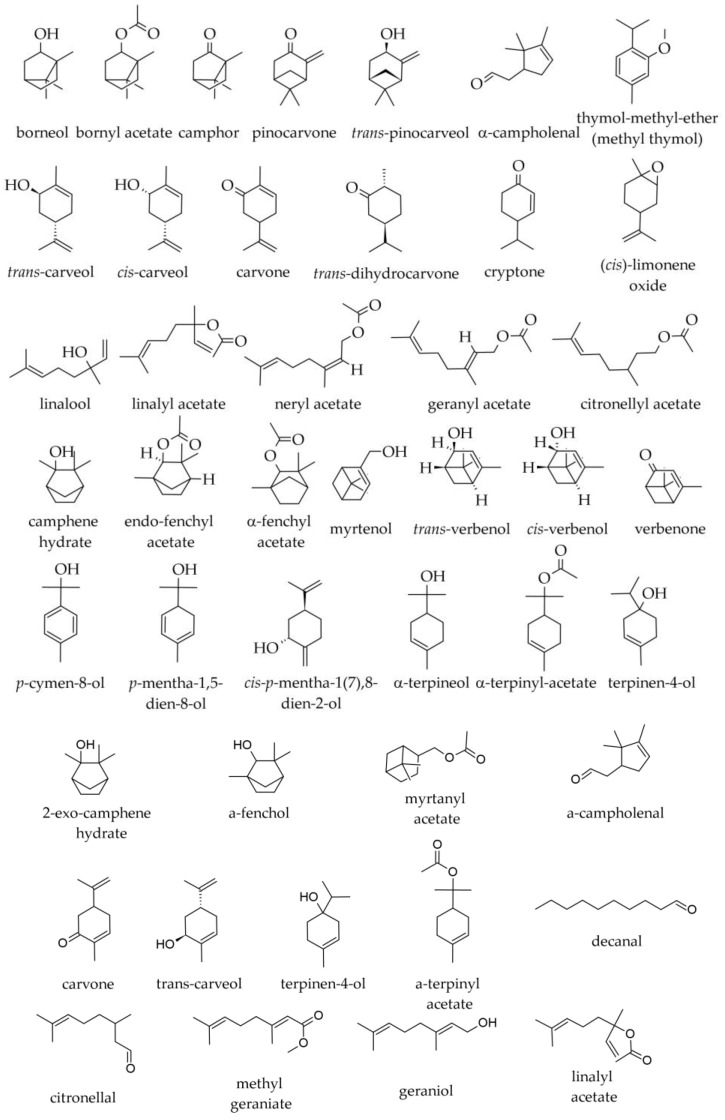
Oxygenated monoterpenes identified in essential oils of various parts of *Abies alba* Mill.

Among oxygenated monoterpenes and other non-terpenic oxygenated compounds (Figure 4), the following were found in specimens from the Southern Carpathians and the Balkan Peninsula/South Balkans: bornyl acetate (1.9–8.8%); borneol (0.9–2.8%); valencene (0.0–1.8%); α-terpinyl acetate (traces—0.4%); α-terpineol (traces—0.3%); geranyl acetate (traces—0.2%); (E)-2-hexenal (0.1%); linalool (traces—0.1%); camphene hydrate (traces—0.1%); linalyl acetate (traces—0.1%); methyl thymol (i.e., thymol methyl ether, 0.0–0.1%); neryl acetate (0.0–0.1%); terpinen-4-ol (traces—0.1%); and, in traces only, camphor, δ-3-carene, citronellol, cyclohexanol, eucalyptol, α-fenchol, 2-heptyl acetate, hexanal, hexanol, (Z)-3-hexenol, *cis*-*p*-menth-2-ene-1-ol, *trans*-*p*-menth-2-ene-1-ol, 2-nonanone, and *cis*-pinocamphone [2,36,39]. In a sample of commercial origin from Germany, α-terpinyl acetate represented 0.5%, whereas, in the essential oil prepared with needles from the Botanical Garden of the University of Würzburg, it was not detected [38]. 

Similar compositions to those presented above, with large quantitative variations, were reported in samples of other origins, the season often not being mentioned. In a sample from Slovakia, α-pinene was the leading compound, present in amounts higher than β-pinene [40]. In a sample from Italy and a commercial sample from Germany, limonene was the leading compound (32.5–34.08%), followed by α-pinene (30.8–31.66%), camphene (5.76–11.2%), β-pinene (2.99–7.5%), β-caryophyllene (4.21–5.8%), and bornyl acetate (1.29–4.2%) [37,38]. The following monoterpene hydrocarbons were reported in various samples: α-pinene (2.13–30.8%); β-pinene (0.5–32.8%); α-limonene (6.1–54.74%); camphene (5.76–19.81%); tricyclene (0.52–12.90%); α-fenchene (0.0–2.6%); santene (0.01–5.00%); *o*-cymene (0.0–1.5%); β-phellandrene (traces—4.9%); γ-terpinene (traces—1.1%); δ-3-carene (0.0–13.85%); β-myrcene (0.6–1.86%); α-terpinolene (0.3–0.5%); 2-bornene (bornylene, 0.0–0.2%); *p*-cymene (0.0–0.57%); α-terpinene (0.0–1.24%); and in traces, if not completely absent, (Z)-β-farnesene, β-fenchene, pseudolimonene, α-phellandrene, and sabinene [35,37,38,40]. In a Korean sample, δ-3-carene and tricyclene had unusually high levels (13.85% and 12.90%, respectively) [35] compared with a large number of European samples, where they had much lower values (under 3.5%). 

Oxygenated monoterpenes reported in samples of other origins were bornyl acetate (0.96–9.0%), α-terpineol (0.2–0.6%), citronellyl-acetate (0.0–0.4%), camphor (0.0–0.2%), endo-fenchyl acetate (0.2%), *cis*-limonene oxide (0.2%), *trans*-pinocarveol (traces—0.2%), borneol (0.1%), α-campholenal (0.0–0.1%), cryptone (0.0–0.1%), pinocarvone (0.0–0.1%), and in traces, carvone, *p*-cymen-8-ol, myrtenol, 4-terpinenol, *trans*-verbenol, and verbenone [34,37,38]. 

A commercial sample from a German company (Farfalla Essential AG) reported the following monoterpenes, but neither the season nor even the plant parts from which the oil was derived were specified in the source paper: δ-limonene (40.0%); α-pinene (32.8%); (+)-camphene (6.48%); β-pinene (2.82%); santene (1.20%); and myrcene (1.09%) [41].

**Figure 5:**
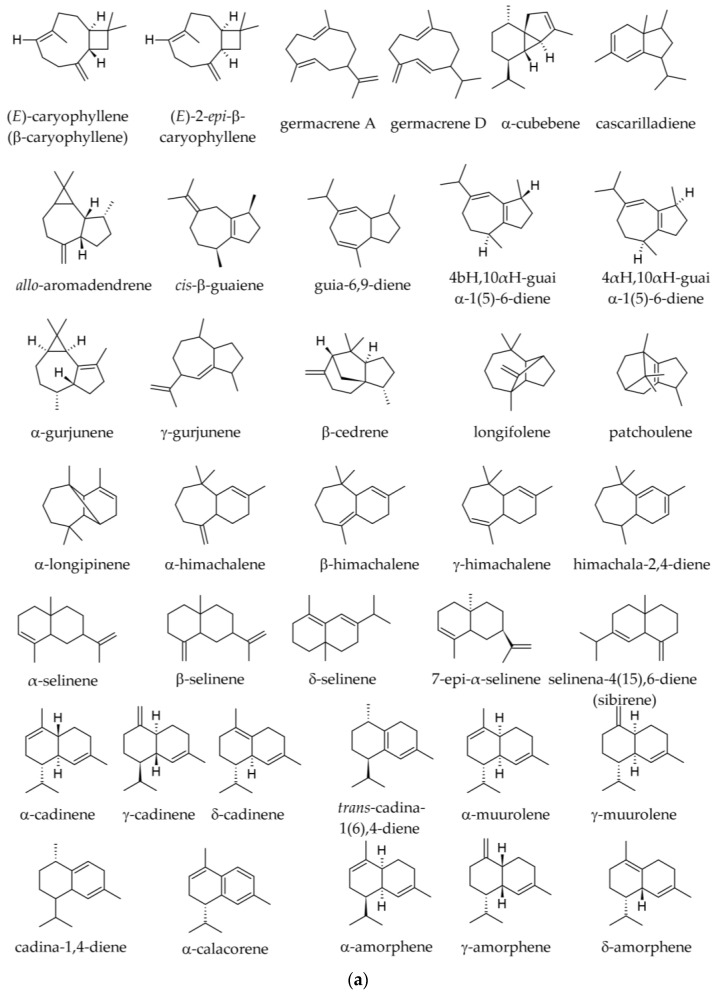

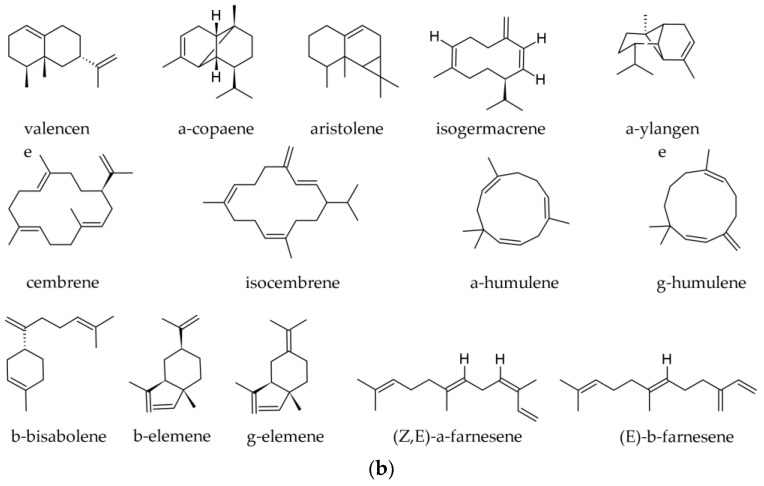
(**a**) Main sesquiterpene hydrocarbons identified in essential oils of various parts of *Abies alba* Mill. (**b**) Sesquiterpene hydrocarbons identified in essential oils of various parts of *Abies alba* Mill.

The variation of sesquiterpene hydrocarbons (Figure 5) by season in samples obtained from Albania is shown in Table 3. In those samples, E-caryophyllene and α-humulene were present in all populations and positively correlated. The 10-*epi*-γ-eudesmol varied widely among populations and tended to increase from north to south [19]. In a Korean sample, the following sesquiterpene hydrocarbons were reported: caryophyllene (2.18%); santene (bicyclo [2.2.1]hept-2-ene,2,3-dimethyl) (1.64%); β-elemene (0.72%); humulene (0.20%); (E)-β-farnesene (0.0–0.2%); valencene (0.13%); α-bisabolene (0.12%); and aromadendrene (0.05%) [35].

The following sesquiterpene derivatives (mostly hydrocarbons) were reported in specimens from the Southern Carpathians and the Balkan Peninsula (summer samples): (E)-β-caryophyllene (0.9–5.7%); δ-cadinene (traces—1.7%); globulol (0.0–0.5%); germacrene D (traces—0.9%); γ-cadinene (traces—0.8%); 10-*epi*-γ-eudesmol (0.0–0.8%); α-selinene (traces—0.6%); γ-elemene (0.0–0.5%); α-ionone (0.0–0.4%); longifolene (traces—0.4%); β-elemene (0.0–0.3%); α-himachalene (traces—0.3%); humulene (0.2–2.6%); aristolene (0.0–0.7%); δ-amorphene (traces—0.3%); β-eudesmol (0.0–0.3%); γ-gurjunene (0.0–0.3%); β-himachalene (traces—0.3%); β-dihydro-agarofuran (traces—0.2%); α-copaene (traces—0.2%); γ-himachalene (traces—0.2%); α-muurolene (traces—0.2%); γ-muurolene (0.0–0.2%); sibirene (traces—0.2%); α-ylangene (traces—0.2%); himachala-2,4-diene (0.1–1.0%); α-longipinene (0.1–0.6%); β-selinene (0.1%); *trans*-cadina-1(6),4-diene (0.0–0.1%); α-cadinene (0.0–0.1%); caryophyllene oxide (0.0–0.1%); α-muurolol (0.0–0.1%) 7-*epi*-α-selinene (0.0–0.1%); and in traces, α-amorphene, cyclosativene, 6,9-guaiadiene, longicyclene, *cis*-muurola-3,5-diene, β-selinene, δ-selinene [2,36,39]. The diterpene (5.9-1 O)-kaur-l5-ene was reported in traces [36]. In samples from other origins the following sesquiterpene hydrocarbons were reported: (E)-caryophyllene (β-caryophyllene, 2.78–7.0%); β-himachalene (0.0–2.59%); α-himachalene (0.00–1.10%); α-longipinene (0.0–0.9%); longifolene (0.1–0.6%); γ-humulene (0.0–1.1%); α-humulene (0.2–2.02%); δ-cadinene (0.0–0.6%); α-gurjunene (0.0–0.4%); γ-muurolene (0.0–0.3%); α-selinene (0.0–0.1%); and in traces, α-amorphene, β-bisabolene, α-cadinene, α-copaene, and α-muurolene, β-selinene (traces) [34,37,40,41].

**Figure 6:**
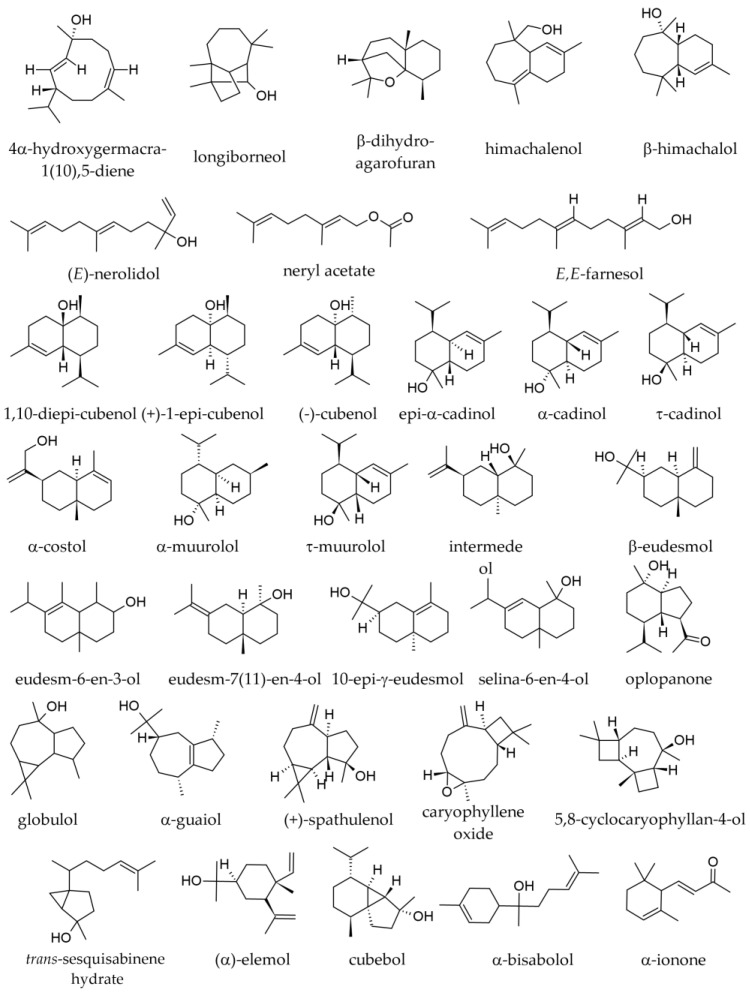
Oxygenated sesquiterpenes identified in essential oils of various parts of *Abies alba* Mill.

In Albanian samples collected from different seasons, oxygenated sesquiterpenes (Figure 6) tend to be less represented in the winter samples than in the summer ones (Table 4). Only caryophyllene oxide (0.2–1.0%) was reported as an oxygenated sesquiterpene in Slovakian, Italian, and French samples [34,34,37,38,40].

In an analysis of 16 samples of needle essential oils of commercial origin, the following ten compounds had the highest concentrations: limonene (6.1–54.7%); α-pinene (0.5–2.8%); β-pinene (7.4–31.7%); camphene (5.8–17.3%); bornyl acetate (0.4–14.2%); β-phellandrene (0.01–4.9%); β-caryophyllene (0.1–4.2%); tricyclene (0.5–2.6%); myrcene (0.7–2.5%); and α-terpineol (0.07–2.3%) [42]. A comparison with the data above indicates that these 16 samples are not representative of the wide variations that can be encountered when compared with other commercial or non-commercial samples. Therefore, specifying only the name and source of essential oil, in the absence of minimal information on the leading ingredients, is of little use in understanding the nature of essential oil.

Usually, chemical analyses of essential oils report many compounds in traces, which are believed to be caused by several factors. These include incomplete suppression of recessive genes, low activity of enzymes that are not specific, and chemical artifacts that may occur during the extraction and isolation processes. For these reasons, in detecting chemical profiles, some authors ignore compounds present in traces only [19].

Besides mono- and sesquiterpenes, which constitute the quasi-totality of essential oils, a few non-terpenic compounds were reported in the essential oil obtained from leaves and twigs, 4-hydroxy-4-methyl-2-pentanone (0.06%) and androstan-17-1,3-ethyl-3-hydroxy-,(5a) (0.12%) (but the identification was only based on mass spectra) [35].

#### 3.1.2. Essential Oil from Twigs and Branches

E. Duquesnoy et al. (2007) analyzed 53 different samples of essential oil obtained by hydrodistillation from twigs collected from six Corsican forests. The essential production yield, estimated on a fresh basis (*w*/*w*), varied between 0.10 and 0.26% depending on the harvesting location. The lowest yields were reported for samples from Carbini (0.10–0.13%), while the highest yields for samples from Rospigliani (0.13–0.26%). Among them, the authors selected two samples with very different chromatographic profiles and identified 65 chemical compounds, covering the large majority of the oil compositions (98.1% and 95.4%, and 44 and 52 chemical compounds, respectively) [1]. Both oils contained mostly monoterpene hydrocarbons (90.8% and 85.0%, respectively), with significantly less oxygenated monoterpenes (5.1% and 6.3%); sesquiterpenes only represented 1.8% and 3.2%, respectively, with the large majority being hydrocarbons and a small proportion being oxygenated (1.6% + 0.2% and 2.% + 0.7%, respectively, for the two samples). Based on principal component analysis applied to the 53 samples, the authors identified two clusters, covering 64% and 36% of all samples, respectively. The first cluster was distinguished by a high level of limonene (mean 46.1% ± 8.1), as well as camphene (16.9% ± 4.6) and α-pinene (12.2% ± 4.3). The second cluster was distinguished by high levels of camphene (mean 23.7% ± 5.0), α-pinene (18.5% ± 9.8), limonene (15.6% ± 7.9), β-phellandrene (23%), and β-pinene (12%). The investigators found no relationship between the cluster membership and the geographic origin of the samples. Essential oils dominated by limonene were also reported in samples from France, Austria, and Greece, whereas oils belonging to cluster II seem not to have been reported previously [1].

The following monoterpene compounds were reported in the two samples selected by Duquesnoy et al. (2007) among the 53 samples of Corsican origin, based on their different chromatographic profiles: limonene (9.3–68.3%); camphene (9.1–20.6%); α-pinene (6.4–19%); β-phellandrene (0.4–15.1%); β-pinene (0.8–11.6%); santene (1.3–4.3%); bornyl acetate (2.3–2.7%); tricyclene (1.1–3.0%); myrcene (1.0–2.2%); citronellyl acetate (0.5–1.5%); α-terpineol (0.3–0.8%); citronellol (0.2–0.5%); terpinolene (0.0–0.8%); geranyl acetate (0.0–0.5%); linalool (0.2–0.3%); decanal (capric aldehyde, 0.1%); 2-exo-camphene hydrate (0.0–0.2%); carvone (0.0–0.2%); citronellal (0.0–0.2%); α-phellandrene (0.0–0.2%); borneol (0.0–0.1%); α-campholenal (0.1%); terpinen-4-ol (0.1%), *trans*-carveol (0.0–0.1%); α-fenchol (0.0–0.1%); geraniol (0.0–0.1%); methyl geraniate (0.0–0.1%); linalyl acetate (0.0–0.1%); myrtanyl acetate (0.0–0.1%); α-terpinene (0.0–0.1%); α-terpinyl acetate (0.0–0.1%); *p*-cymene (traces—0.1%); γ-terpinene (traces—0.1%). Sabinene, 1,8-cineole, (E)-β-ocimene, and *p*-cymenene were only detected in traces or were completely absent from some samples [1].

The following sesquiterpenes were reported in the twig essential oils: (E)-β-caryophyllene (0.4–0.7%); (E)-β-farnesene (traces—0.4%); α-longipinene (traces—0.3%); β-himachalene (0.2%); junipinene (0.2%); δ-cadinene (0.1–0.3%); longiborneol (0.1–0.3%); longifolene (0.0–0.3%); α-humulene (0.1–0.2%); cembrene (traces—0.2%); γ-humulene (0.0–0.2%); β-bisabolene (0.0–0.1%); α-cadinol (0.0–0.1%); τ-cadinol (0.0–0.1%); (E)-2-*epi*-β-caryophyllene (0.0–0.1%); β-cedrene (0.0–0.1%); isocembrene (0.00–0.1%); cubebol (0.0–0.1%); (Z,E)-α-farnesene (0.0–0.1%); himachalenol (0.0–0.1%); τ-muurolol (0.0–0.1%). Longicyclene, β-elemene, *trans*-calamenene, caryophyllene oxide, and manoyl oxide were only reported in traces or absent from some samples [1]. We were not able to find any chemical representation for “junipinene” or any synonyms.

Dodecanal was reported in amounts of 0.2–0.4%. Isopimaradiene was the only diterpene detected in quantifiable amounts (0.0–0.1%); diterpenes were detected in traces only in one sample and in a proportion of about 0.4% in a second sample [1].

#### 3.1.3. Supercritical Fluid Extracts from Twigs

Supercritical fluid extracts (SFE) obtained from twigs have considerable differences compared with essential oils obtained from the same plant parts, as illustrated by an analysis of two samples belonging to the two Corsican clusters mentioned above. In both SFE samples, the proportion of monoterpene hydrocarbons is lower than in the essential oil, and in certain cases, they are completely absent. For instance, limonene was 43.5% of the essential oil in the first sample but only 17.5% in the corresponding SFE sample; it was 15.6% in the second sample and only 5.6% in the corresponding SFE sample. α-Pinene represented 18.0% and 11.4% of the essential oil samples but only 3.4% and 2.6% of the SFE samples. Similarly, camphene concentrations diminished from 13.7 and 15.7% in the essential oils to only 1.9% and 3.9% in SFE, respectively. Regardless of its concentration in the essential oil, in the SFE, the β-phellandrene concentration was about half that in the essential oil (14.4% in the essential oil, 7.0% in SFE, 0.2% in the essential oil, and 0.1% in SFE). Santene, found in the two essential oils in 3.6% and 3.0% concentrations, was found in 0.1% or only the traces in the SFEs [43]. α-Terpineol, detected in low amounts in the essential oils (0.7%, 1.2%), is completely undetected in SFEs [43].

Conversely, sesquiterpene compounds are extracted much more efficiently in SFE, so in essential oils, they represented 4.1% and 3.0%, but 27.3% and 30.0% in SFE. Moreover, a number of 11 sesquiterpene hydrocarbons were only detected in SFE (in proportions varying between 0.2% and 5.2%), while being absent in the essential oil, which indicates that not all sesquiterpene hydrocarbons may be extracted in the essential oils; the 11 molecules were mostly selinan, cadinene, and himachalane derivatives [43].

The following monoterpene hydrocarbons were detected in SFEs: limonene (5.6– 17.7%); β-phellandrene (0.1–7.0%); camphene (1.9–3.9%); α-pinene (2.6–3.4%); β-pinene (0.4–2.7%); tricyclene (0.3–0.5%); myrcene (0.3–0.4%); santene (traces—0.1%) [43]. The following monoterpene oxygenated derivatives were reported in SFEs: bornyl acetate (1.0–8.1%); geranyl acetate (0.0–1.7%); citronellyl acetate (0.7–1.1%); borneol (0.0–0.9%); linalyl acetate (0.0–0.4%), geraniol (0.0–0.3%), terpinolene (0.1%), citronellol (0.0–0.1%), and decanal (0.0–0.1%). The following sesquiterpene hydrocarbons were reported in SFEs: (E)-β-caryophyllene (5.6–6.3%); β-selinene (3.8–5.2%); himachala-2,4-diene (2.4–2.6%); α-longipinene (2.3%); α-humulene (2.1–2.2%); γ-humulene (1.6–2.0%); δ-cadinene (0.7–1.8%); longifolene (1.3–1.7%); β-himachalene (0.9–1.3%); α-selinene (0.7–1.1%); γ-curcumene (0.4–1.0%); α-himachalene (0.7–0.9%); γ-himachalene (0.2–0.8%); *ar*-himachalene (0.4–0.7%). Apparently, longiborneol (0.0–2.6%) was the only oxygenated sesquiterpene in SFEs. The diterpene *cis*-abienol was also only detected in SFE, in notable amounts (17.3% and 7.5% of the two SFEs), but not in the two essential oils, probably due to its lack of volatility [43].

#### 3.1.4. Bark Oleoresin and Bark Triterpenoids

Many conifers have evolved resin ducts and are capable of storing important amounts of oleoresin when the plant is wounded or damaged [44], and *A. alba* makes no exception to this rule. The chemical composition of the bark oleoresin is different from that of the needle oleoresin. In the Albanian samples (see above, Section 3.1.4), a number of 33 compounds were identified, corresponding to 95–97% of the essential oil. Similarly to the needle essential oil, the winter samples tended to have a richer composition (a greater number of compounds) than the summer samples [19].

The essential oil consists primarily of monoterpene hydrocarbons (64.2–85.8%), whereas oxygenated monoterpenes tend to represent (on average) less than 2% of the oil. The monoterpene hydrocarbons identified, with their proportions in the winter and summer samples, are shown in Table 5. In Albanian samples, an increasing trend for limonene content from north to south was reported, whereas a reverse trend was reported for camphene. Significant positive correlations were reported between α-pinene and β-pinene (possibly common biosynthetic origin), whereas significant negative correlations were reported between α-pinene and limonene, as well as for β-pinene and limonene [19].

Sesquiterpene hydrocarbons represented 14.8–30.8% of the essential oils prepared from winter samples and 13.5–18.0% of the essential oils prepared from summer samples. The sesquiterpene hydrocarbons identified, with their proportions in the winter and summer samples, are shown in Table 6. A robust positive correlation was reported for E-caryophyllene and α-humulene, two compounds known to have a common biosynthetic origin, and such a correlation was also reported in other gymnosperms [19].

**Figure 7:**
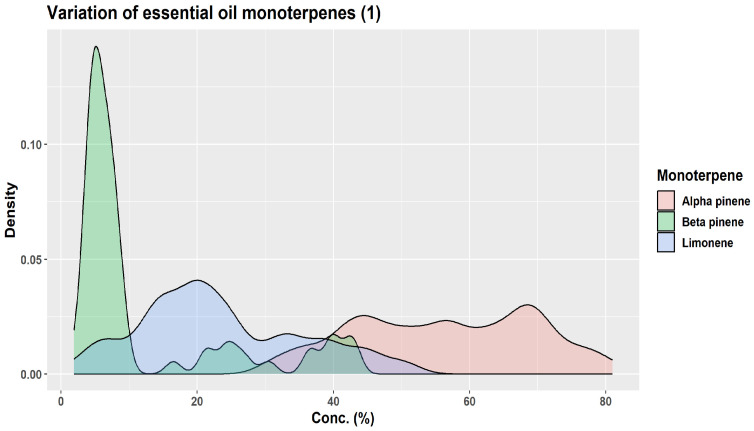
Variation of α-pinene, β-pinene, and limonene contents in 63 provenances of *A. alba* bark essential oil (Source data [45]).

**Figure 8:**
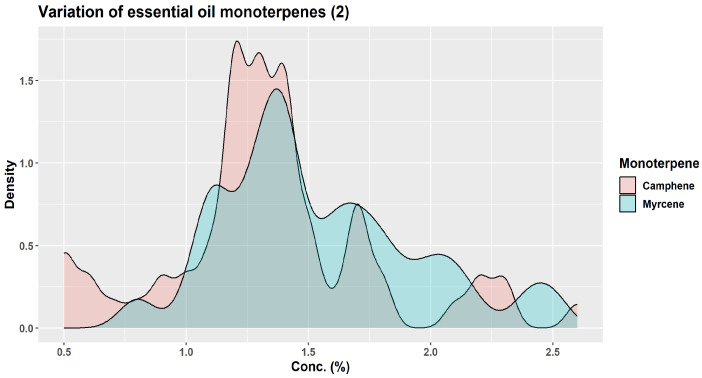
Variation of camphene and myrcene contents in 63 provenances of *A. alba* bark essential oil (Source data [45]).

**Figure 9:**
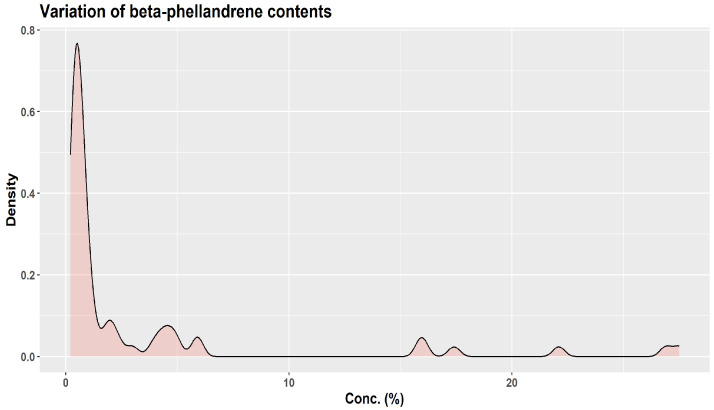
Variation of β-phellandrene contents in 63 provenances of *A. alba* bark essential oil (Source data [45]).

About three decades ago, KJ Lang studied the chemical variability of a large number (over 1500) of oleoresin samples obtained from two-year-old twig cortex of *A. alba* originating from 63 distinct sources in Europe [1,45]. Using unsupervised cluster analysis, KJ Lang distinguished three “provenance groups”: group I (Bavarian Forest, Eastern Alpine foothills, and certain areas of the Alps); group II (Western Alpine foothills, the Alps, the Black Forest, France, Northern Italy, and zones of Eastern Europe); and group III (Central and Southern Italy plus Balkan countries) [45]. Based on the quantitative variation of six monoterpenes only (α-pinene, β-pinene, limonene, camphene, myrcene, β-phellandrene), the authors indicated the presence of 13 chemotypes and four major groups, depending on the European region of origin: Western–Central, Eastern–Central, Southern–Eastern, and Central–Southern [1]. Their results are particularly interesting in showing how wide the variations in the six monoterpenes can be (Figure 7, Figure 8 and Figure 9) [45].

**Figure 10:**
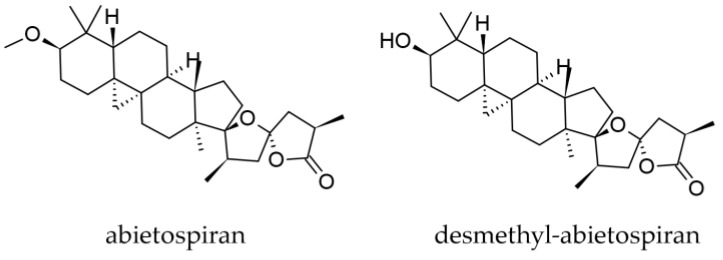
Triterpenes identified in the bark of *Abies alba* Mill.

Besides the rich mono- and sesquiterpene profile in oleoresin, two triterpenoids have been isolated from the bark and identified up to now, abietospiran and desmethylabietospiran ((23S,-25R)-3α-hydroxy-17,23-epoxy-9,19-cyclo-9β-lanostan-26,23-olide) (Figure 10) [46,47].

#### 3.1.5. Cone Essential Oils

Cones (cone scales) are poorer in essential oil than seeds, with an oil yield of around 0.5–0.75% for *A. alba* and 0.5% for *A. koreana* E.H. Wilson [33,48]. The essential oil prepared by hydrodistillation from the cones of *A. alba* contains a similar proportion of total monoterpenes (94.4%) as the seeds (93.6%), but in the cones, the proportion of oxygenated monoterpenes is increased compared with the seeds (8.0% vs. 0.2) [33].

The leading ingredients of the cone essential oil are, as mentioned above, monoterpenes: α-pinene (50–57.1%), limonene (10.1–27.2%), β-pinene (6.5–9.4%), and verbenone (2.5–6.4%) [33]. Other monoterpenes reported in smaller amounts in the cone essential oils are β-myrcene (0.3–0.7%), *trans*-pinocarveol (0.7–3.2%), *trans*-verbenol (0.7%), α-campholenal (0.6%), camphene (0.3–0.5%), *trans*-carveol (0.5%), thuja-2,4(10)-diene (dehydrosabinene, 0.5%), α-fenchene (0.0–0.4%), borneol (0.3–1.4%), α-campholenal (0.3%), limonene oxide (0.3%), *p*-mentha-1,5-dien-8-ol (0.3%), myrtenol (0.3–1.0%), α-terpineol (0.3%), *cis*-carveol (0.2%), *p*-cymene (0.2–0.4%), carvone (0.0–0.3%), α-phellandrene (traces—0.2%), pinocarvone (0.2%), sabinene (traces—0.2%), *cis*-verbenol (0.2–0.8%), bornyl acetate (traces—0.1%), *trans*-dihydrocarvone (carvomenthone, 0.1%), m-cymene (0.1%), *p*-cymen-8-ol (0.1%), globulol (0.0–0.1%), *cis*-*p*-mentha-1(7),8-dien-2-ol (0.1%), terpinolene (0.1%), and tricyclene (traces—0.1%) [33,48]. The following were only reported in traces (or absent in some samples): camphor; δ-car-3-ene; *p*-cymenene; linalool; *p*-mentha-1,3,8-triene; *cis*-*p*-mentha-2,8-dien-1-ol; *trans*-*p*-mentha-2,8-dien-1-ol; *trans*-*p*-menth-2-en-1-ol; (E)-β-ocimene; β-phellandrene; pinocamphone; santene; γ-terpinene; terpinen-4-ol; and α-thujene [33,48]. Analysis of the essential oils emitted by cones collected in France indicated a similar composition with respect to the main ingredients in α-pinene (57.2%), limonene + β-phellandrine (27.7%) (the two monoterpenes were not separated in the GC analysis), β-pinene (8.5%), myrcene (3.3%), camphene (1.3%), and in traces tricyclene and sabinene [32].

Sesquiterpene hydrocarbons are less represented in cones than in seeds (2.0 vs. 5.20), but oxygenated sesquiterpenes are also slightly higher in cone essential oil than in that obtained from seeds (1.5% vs. 1.0%) [33]. The leading sesquiterpenes are selin-6-en-4-ol (0.6%), longifolene (traces—0.4%), δ-cadinene (0.1–0.3%), γ-cadinene (0.2%), α-cadinol (traces—0.2%), (E)-β-caryophyllene (0.1–0.2%), himachala-2,4-diene (0.2%), α-humulene (traces—0.2%), α-amorphene (0.1%), α-cadinene (0.1%), τ-cadinol (0.0–0.1%), β-caryophyllene oxide (0.0–0.1%) α-copaene (traces—0.1%), α-cubebene (0.1–0.3%), 1-epicubenol (cubenol, 0.1%), β-elemene (traces—0.1%), elemol (traces—0.1%), α-guaiol (0.0–0.1%), β-himachalol (traces—0.1%), intermedeol (0.1%), α-longipinene (traces—0.1%), τ-muurolol (0.0–0.1%), α-muurolene (0.1%), patchoulene (0.0–0.1%), β-selinene (0.1%), and δ-selinene (0.1%) [33,48]. Agarospirol, γ-amorphene, δ-amorphene, *allo*-aromadendrene, bicycloelemene, β-bisabolene, 7αH,10βH-cadina-1(6),4-diene, cadina-1,4-diene, α-calacorene, β-calacorene, *cis*- or *trans*-calamenene, calarene, 2-*epi*-(E)-β-caryophyllene, caryophyllene oxide, cascarilladiene, α-cedrene, 1,10-diepicubenol, epizonarene, α-fenchol, germacrene D, 4α-hydroxygermacra-1(10),5-diene, guaia-6,9-diene, 4αH,10αH-guaia-1(5)-6-diene, β-himachalene, γ-himachalene, humulene oxide, longiborneol, β-longipinene, 13-*epi*-manoyl oxide, γ-muurolene, sativene, selina-4(15),6-diene (sibirene), spathulenol, and α-ylangene were reported in traces or absent from some samples [33,48].

**Figure 11:**
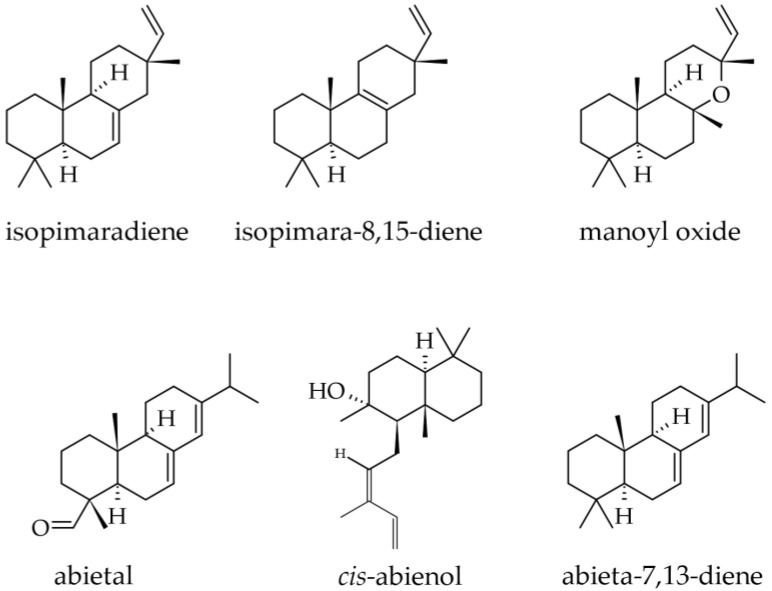
Diterpenes identified in *Abies alba* Mill.

Cone essential oil was the only one that included small but quantifiable amounts of diterpenes, both hydrocarbons (0.2%) and oxygenated (0.2%). They consist of abietal (0.0–0.2%), manoyl oxide (0.0–0.2%), abieta-7,13-diene (0.1%), isopimara-8,15-diene (0.1%) (Figure 11), whereas abieta-8(14),13(15)-diene was only found in traces [33].

The composition of the cone essential oils is similar in *A. alba* and *A. koreana*, with what seem to be minor differences in the leading compounds (50% α-pinene in *A. alba* vs. 56.8% in *A. koreana*; 5.6% β-pinene in *A. alba* vs. 11.2% in *A. koreana*; 27.2% limonene in *A. alba* vs. 13.0% in *A. koreana*) [33]. Different analyses performed on the same two species from the same geographic region in different years indicated consistent results, with small differences (e.g., 57% α-pinene in an assay performed five years earlier for *A. alba*) [48].

#### 3.1.6. Seed Essential Oils

The seeds were reported to be very rich in essential oils (7.4%, 12.4%, 14.3%—depending on origin and time of collection), their contents being in one publication from Poland about three times higher than the contents in essential oils of *A. koreana* E.H.Wilson seeds (about 3.8%) [33,48]. The amount of essential oil seems to be also slightly higher in the seeds of *A. alba* than in those of *A. pinsapo* subsp. *marocana* (Trab.) Emb. and Maire (syn. *A. marocana* Trab.) (5.3% reported in one paper) [48,49] or *A. nordmanniana* (Steven) Spach (6.0%) [48]. The essential oil has a resinous, woodsy, pleasant scent with a discrete note of lemon–orange fragrance that may be related to the presence of limonene [33]. The seed essential oil includes primarily monoterpene hydrocarbons (88% in one sample and over 93% in another, both from different regions of Poland and collected in different years) and sesquiterpenes (around 6%), unlike *A. koreana*, where the second largest group of components consists of oxygenated derivatives (in the essential oil of *A. alba* seed, oxygenated monoterpenes represented only 0.2% and oxygenated sesquiterpenes 1.1%) [33,48].

The leading compound is (−)-limonene (70.1–82.9%); mostly, its levorotatory isomer (over 96% of total limonene) (in absolute terms, (+) limonene was reported as 2.1–2.6%). Other monoterpene ingredients of the seed essential oil are α-pinene (6.3–11.5% in different samples), β-pinene (1–2.1%), β-myrcene (2.8–3.1%), bornyl acetate (0.1%), camphene (0.1% in one sample, only traces in two samples), camphor (0.1% or traces only), α-fenchene (0.1% in two samples, absent in one), terpinolene (0.1%), α-terpinyl acetate (0.1%), tricyclene (0.1% in one sample, only traces in two different samples), verbenone (0.1%, traces or absent) and in traces (or absent from certain samples) *cis*-anethole, *trans*-anethole, apiol, borneol, bornyl acetate, carvacrol, *trans*-carveol, *cis*-carveol, carvone, *trans*-carvone epoxide, citronellol, citronellyl acetate, *p*-cymene, eugenol, fenchone, geranial, geranyl acetate, linalool, menthol, *cis*-*p*-menth-2,8-dien-1-ol, *trans*-*p*-menth-2-en-1-ol, myrtenol, neral, (E)-β-ocimene, α-phelandrene, *trans*-pinocarveol, sabinene, α-terpinene, γ-terpinene, α-terpineol, α-terpinyl acetate, thymol, and *cis*-verbenol [33,48].

Among the sesquiterpenes, the following were reported: δ-cadinene (0.9–1.5%); guaiol (0.0% to 1.5%); spathulenol (0.0% to 1.1%); selin-6-en-4-ol (0.6%); γ-cadinene (0.5–1.0%); (E)-β-caryophyllene (0.5–0.8%); patchoulene (traces—0.7%); *allo*-aromadendrene (0.0% to 0.5%); α-cubebene (from traces—0.4–0.5%); δ-selinene (0.5%); α-amorphene (traces—0.3%); β-selinene (0.3%); β-elemene (0.2–0.3%); β-himachalol (traces—0.3%); 1-epicubenol (traces—0.2%); globulol (0.0% to 0.2%); himachala-2,4-diene (traces—0.2%); α-humulene (0.2–0.7%); isogermacrene D (absence to 0.3%); longifolene (0.2%); α-muurolene (traces—0.2%); γ-amorphene (0.1%); δ-amorphene (0.1%); α-cadinene (traces—0.1%); α-cadinol (traces—0.4%); 2-*epi*-(E)-β-caryophyllene (0.1%); β-caryophyllene oxide (0.0% to 0.1%); cascarilladiene (0.1%); guaia-6,9-diene (0.1%); 4αH,10αH-guaia-1(5)-6-diene (0.1%); 4βH,10αH-guaia-1(5)-6-diene (0.1%); 4α-hydroxygermacra-1(10),5-diene (0.1%); β-himachalene (0.1%); γ-himachalene (0.1%); α-longipinene (traces—0.1%); γ-muurolene (traces—0.1%); selina-4(15),6-diene (0.1%); and α-ylangene (traces—0.1%) [33,48]. The following sesquiterpenes were reported in traces only or were absent from some samples: agarospirol; aromadendrene; (*E*)-*trans*-bergamotol; bicycloelemene; cadina-1,4-diene; cadina-3,5-diene; 7αH,10βH-cadina-1(6),4-diene; τ-cadinol; calacorene; α-copaene; β-cubebene; cubebol; cubenol; 1,10-diepicubenol; *cis*-β-elemene; elemol; epizonarene; eudesma-5,7(11)-diene; farnesal; (*E,E*)-farnesyl acetate; germacrene D; humulene epoxide II; intermedeol; longiborneol; *cis*-muurola-4(15),5-diene; T-muurolol; (E)-nerolidol; sativene; β-selinene; selina-4(15),7-diene; and selina-3,7-diene. Most sesquiterpenes were hydrocarbons (5.2% in total), whereas oxygenated ones were less represented (1.1% in total). While in the essential oil prepared from *A. alba* seeds, limonene represented 82.9%, in the essential oil prepared from *A. koreana* seeds, limonene represented only 53.7%, the latter containing higher amounts of α-pinene, camphene, and bornyl acetate [33]. Analyses performed at a distance of about five years in the same geographic regions showed consistency in the composition, with some quantitative variation (the leading compound, (−)-limonene, was in a proportion of about 70% in the first assay and of 82.9% in the second [33,48].

Manoyl oxide, 13-epi manoyl oxide, abieta-7,13-diene, and abietal were the only diterpenoids detected in the seed essential oil, in traces only [48].

#### 3.1.7. Seed Hydrolates

The hydrolate (floral water, hydrosol) obtained from the waste liquid generated by hydrodistillation of the plant seed is claimed to have a fresh, agreeable, resinous odor. Its content in essential oil was estimated at 10.7 mg/L (the seed hydrolate prepared from the related species *A. koreana* has 37.3 mg/L of essential oils)). The seed hydrolate contained selin-6-en-4-ol (51.7%), β-himachalol (14.5%), τ-cadinol (10.7%), intermedeol (9.8%), 1-epicubenol (2.1%), elemol (1.7%), 1,10-diepicubenol (1.5%), longiborneol (1.0%), eudesm-7(11)-en-4-ol (0.6%), oplopanone (0.5%), 5,8-cyclocaryophyllan-4-ol (0.3%), cubebol (0.2%), eudesm-6-en-3-ol (0.2%), α-bisabolol (0.1%), borneol (0.1%), α-costol (0.1%), (E)-nerolidol (0.1%), *trans*-sesquisabinene hydrate (0.1%), an isomer of *p*-menth-8-ene-1,2-diol (0.1%), α-terpineol (0.1%), verbenone (0.1%), and in traces, bornyl acetate, 8-hydroxylinalool, terpinen-4-ol, and α-terpinyl acetate. Thus, whereas in the seed essential oil, the prominent fraction was represented by monoterpene hydrocarbons, the seed hydrolate was dominated by oxygenated sesquiterpenes (over 95%). Many compounds presented in the hydrolate were absent from the essential oils and the other way around [33].

#### 3.1.8. Wood Terpenoids

**Figure 12:**
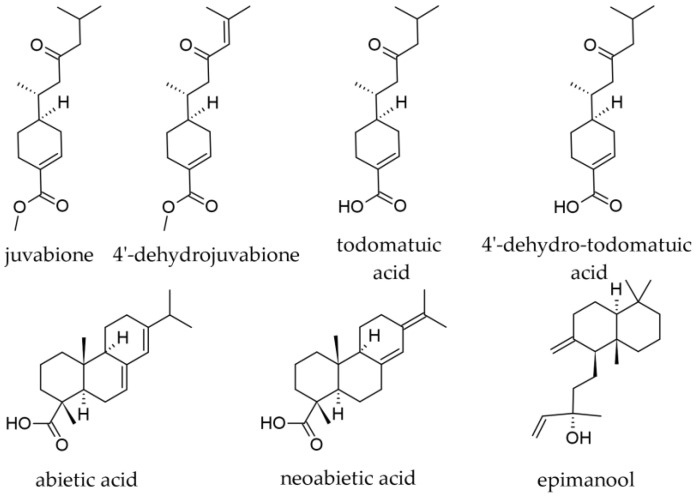
Wood terpenoids identified in *Abies alba* Mill.

Although wood as a plant part of silver fir is not of primary interest for its terpenoid contents, small amounts of terpenoid substances were also reported in it, although their content in terpenoids is not negligible (an extract prepared with ethanol was reported to contain 15.65% terpenes [23]. In a methanolic extract from branch wood, the following terpenoids were identified by LC-DAD–ESI-MS/MS, besides the majority of compounds that belonged to the lignans group: dehydrojuvabione; neoabietic acid; and abietic acid (Figure 12) [50]. In extracts prepared with 100% ethanol from knotwood, the diterpenoid epimanool was also reported [23].

Juvabiones are compounds with strong hormonal activities on juvenile insects and antifungal activity; chemically, they are sesquiterpene acids or non-phenolic sesquiterpene derivatives. In *A. alba* wood, juvabione, 4′-dehydrojuvabione, todomatuic acid, and 4′-dehydrotodomatuic acid were reported, being slightly more abundant in dead knots than in living knots, and two–three times more soluble in hydrophilic solvents (acetone, water) than in lipophilic ones (hexane) [21]. In wood extracts prepared with 100% ethanol, dehydrojuvabione was reported as more abundant than juvabione [23].

### 3.2. Lignans

The bark and wood of *A. alba*, similarly to those of other conifers, are among the richest sources of lignans in the plant world.

#### 3.2.1. Bark Lignans

**Figure 13:**
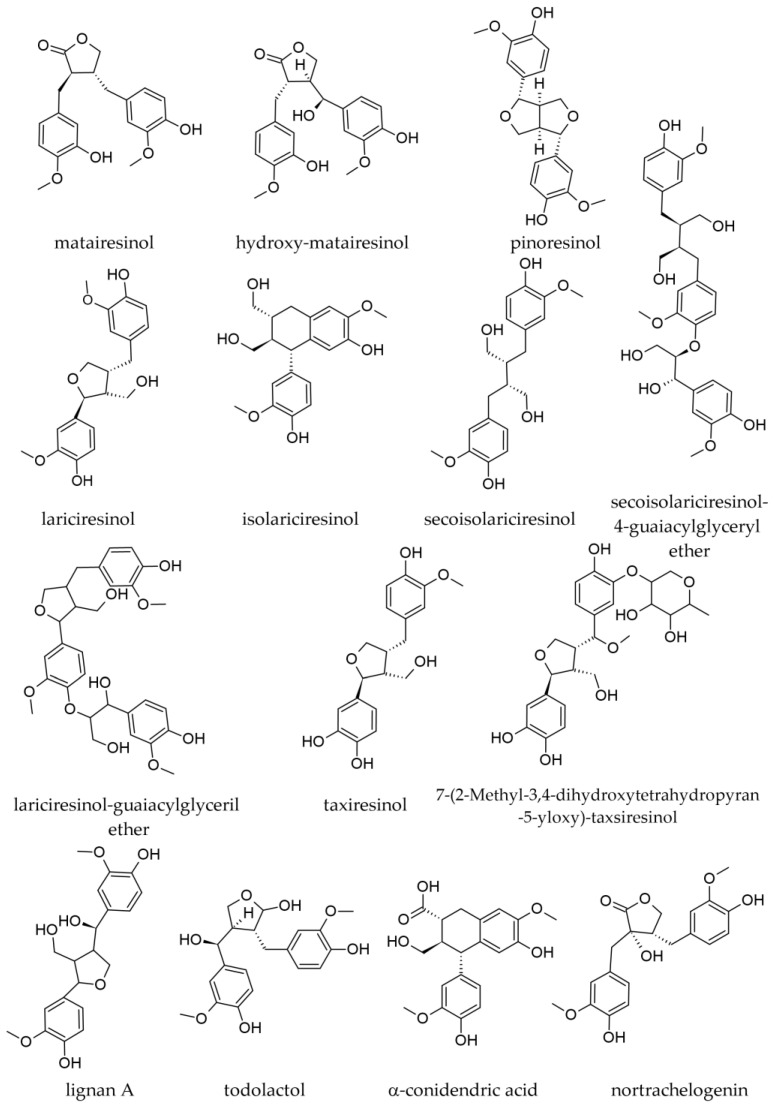
Lignans identified in various parts of *Abies alba* Mill.

The following lignans were reported in extracts prepared with water from bark collected off stems and branches (Figure 13): matairesinol; 7-hydroxymatairesinol (also known as hydroxymatairesinol); lariciresinol; isolariciresinol (cyclolariciresinol); secoisolariciresinol; pinoresinol; lignan A; and oligolignans [18]. Matairesinol seemed to be more abundant in the bark from the stem than in the bark from branches (0.20 mg/g vs. 0.10–0.14 mg/g, d.w.). The trunk bark was several-fold richer in isolariciresinol than the branch bark (0.09 mg/g vs. 0.02 mg/g d.w.), whereas lariciresinol was only present in the trunk bark (0.035 mg/g, d.w.) being absent in the bark from branches. Secoisolariciresinol was also more abundant in the trunk bark than in the branch bark (0.04 mg/g vs. 0.02–0.03 mg/g, d.w.) [18].

In the Abigenol^®^ extract, four lignans were reported, as identified by mass spectrometry: 7-(2-methyl-3,4-dihydroxytetrahydropyran-5-yloxy)-taxiresinol; taxiresinol; secoisolariciresinol; and lariciresinol [4].

#### 3.2.2. Wood Lignans

Extracts prepared from branch-derived wood have been shown to be rich in lignans, besides their contents in phenolic derivatives and carbohydrates [26]. In a methanolic extract from branch wood, the following lignans were identified by LC-DAD–ESI-MS/MS: 7-hydroxylariciresinol; todolactol; isolariciresinol; α-conidendric acid; 7-hydroxymatairesinol; secoisolariciresinol; secoisolariciresinol guaiacylglyceryl ether; lariciresinol; lariciresinol guaiacylglyceryl ether; nortrachelogenin. The most abundant appeared to be secoisolariciresinol and lariciresinol (Figure 13). For nortrachelogenin, the same paper stated its presence in a table but negated it in the discussion section (“we did not observe any signal at expected retention times”; therefore, its presence in *A. alba* is not certainly established) [50].

Vek et al. (2021) reported isolariciresinol in amounts of up to 46.9 mg/g in various wood samples, the richest being the dead knots; lariciresinol was found in amounts up to 32.3 mg/g in wood samples, the highest being found in branch wood. In various wood samples, secoisolariciresinol was better represented, with levels of 37.6 mg/g in branch wood [22]. Another paper reported that secoisolariciresinol represented 26.6% of all compounds in a knotwood acetone extract [51]. Pinoresinol was less abundant in wood, with the highest levels being reported for dead knots (2.5 mg/g). Among wood samples, matairesinol had the highest abundance in branch wood (10 mg/g), with slightly lower levels also found in living and dead knots [22].

A decrease in the contents of various lignans was reported in branches (which are mostly wood, although, apparently, they were milled together with bark) as the distance of the branch from the trunk increased (such a decrease was stronger than the one reported for phenolics in the branches of the same species) [13]. The most abundant lignan was secoisolariciresinol, with levels of 5.95 ± 1.41 mg/g near the trunk and only 0.29 ± 1.03 mg/g at 80 cm away from the trunk. The concentration of isolariciresinol was 1.34 mg/g near the trunk but only 0.14 ± 0.15 mg/kg at 80 cm away. The concentration of several lignans, including lariciresinol, 7-hydroxymatairesinol, pinoresinol, and matairesinol, decreased significantly across the branch length. The concentration of lariciresinol decreased from 1.34 ± 0.19 to 0.15 ± 0.06 mg/kg across the branch length, the level of 7-hydroxymatairesinol from 0.54 ± 0.11 to 0.24 ± 0.30 mg/kg, the level of pinoresinol from 0.49 ± 0.12 to 0.06 ± 0.04 mg/kg, and the contents of matairesinol declined from 0.32 ± 0.08 to 0.05 ± 0.06 mg/kg. A sample of milled branches used for the industrial manufacturing of a silver fir extract (brand name Belinal^®^) analyzed for comparison contained 0.50 mg/kg secoisolariciresinol, 0.26 mg/kg isolariciresinol, 0.21 mg/kg 7-hydroxymatairesinol, 0.17 mg/kg lariciresinol, 0.09 mg/kg pinoresinol, and 0.04 mg/kg matairesinol [13].

In a commercial branch extract (Belinal^®^), the following lignans were identified and quantified: secoisolariciresinol (5.51%); isolariciresinol (1.78%); 7-hydroxymatairesinol (0.89%); lariciresinol (0.79%); matairesinol (0.50%); and pinoresinol (0.24%) [52].

Aqueous extracts prepared from the knotwood of the species contain notably higher amounts of extracted substances than those prepared from stemwood or heartwood [21,22]. Thus, it was reported that in various *Abies* sp., the knotwood might contain 20–50 times more lignans than the stemwood, but large variations are possible not only from species to species but also intraspecifically, from tree to tree. In sapwood and heartwood of *A. alba*, lignans are present in levels of the order of μg/g, whereas, in living or dead knots, they are in levels of the order of mg/g [21].

Extracts prepared with 100% ethanol from knotwood are also very rich in lignans (about 55% of the chromatogram area). Chromatographically, at least 81 peaks were observed, among which 21 compounds were identified. The most important was secoisolariciresinol (the dominant lignan), followed by lariciresinol, 7-hydroxymatairesinol, as well as important amounts of D-pinitol. Secoisolariciresinol, the main lignan of *A. alba wood* (33.40% of the total chromatogram area), has maximal concentrations in the knots located around the crown base, its levels decreasing in the knots positioned toward the stem base or toward the top of the tree. Lariciresinol (9.87% of the total chromatogram area) did not have a similar dependence on the knot position on the tree. Hydroxymatairesinol yield (mg/g) tends to decrease with increasing knot height. Isolariciresinol was reported in smaller amounts in the ethanol extract [23].

The extraction yield seems to be higher in the case of dead knots than of living knots with respect to the total amount of lignans (53–55 mg/g vs. 67–70 mg/g), and some qualitative differences exist between lignans in the two categories of knots [21,22]. The total content of secoisolariciresinol seems to be similar between living knots and dead knots, but in the former product, a small part of this lignan is in the form of dimethyl or monomethyl ether (29–30 mg/g secoisolariciresinol + 0.56–0.57 mg/g dimethyl ether + 2.3 mg/g monomethyl ether vs. 32–36 mg/g). Lariciresinol is present in almost double amounts in dead knots than in living knots (9.9–10.0 mg/g vs. 4.6–5.2 mg/g). Liovil (up to three isomers) is more abundant in living knots than in dead knots (5.5–6.0 mg/g vs. 3.8–4.0 mg/g). The 7-hydroxymatairesinol (4.2 mg/g vs. 4.8–5.2 mg/g), 7-*allo*-hydroxymatairesinol (2.8 mg/g vs. 2.4 mg/g), and matairesinol (2.5–2.6 mg/g vs. 2.6 mg/kg) are present in more or less similar amounts in the two types of knots. Dead knots are richer in isolariciresinol (cyclolariciresinol, 5.7–7.4 mg/g vs. 0.88–0.89 mg/g) and pinoresinol (0.36–0.40 mg/g vs. 0.98–1.0 mg/g) than living knots. Nortrachelogenin was reported in very high amounts in dead knots (270 mg/g), whereas it was absent from living knots. Sesquineolignans (13–16 mg/g), dilignans (12–14 mg/g), and higher oligolignans (2.5–3.1 mg/g) are also present in silver fir knotwood, the dominant ones being dineolignans, with slightly higher amounts in dead knots than in living knots. The lignans are found in *A. alba* in an overwhelming proportion as free aglycones [21].

Although it has generally been reported that lactobacilli could influence the metabolism of certain lignans and drive it to increase the production of lignans, Stojanov et al. (2021) did not find any influence of lactobacilli (10 different species, several of which were derived from human isolates) on the production of enteriodiol or enterolactone from the lignans present in an *A. alba* wood extract. However, the microbial species tested by these authors were different from those in the previously published research, and the lignan levels were lower than those used before [26].

### 3.3. Phenolics

#### 3.3.1. Bark Phenolic Compounds (Including Flavonoids)

A content of about 2.67% (*w*/*w*, d.w.) of total phenols was reported in the bark of the stem and branches of *A. alba* from Slovenia [18]. Hydrophilic extracts obtained from bark contain about 15% total phenols [18]. Instead, on Swiss bark samples extracted using water at 60 °C, a total extraction yield of 10.1–12.0% (*w*/*w*, d.w.) was reported, and total phenolic compounds represented 27.9% of this extract [6,53].

The proportion of phenolic compounds seems to be higher in the bark from the lower parts of the trunk [20]. Although the inner and outer layers of bark have a similar total average of hydrophilic extractives, the phenolic contents differ between the two layers: the inner bark layer was poorer in total phenolics than the outer layer (the latter containing 14.87 mg/g, d.w. more phenolics than the former). The difference could be at least partially due to the phenols from the periderm cork cells [18].

**Figure 14:**
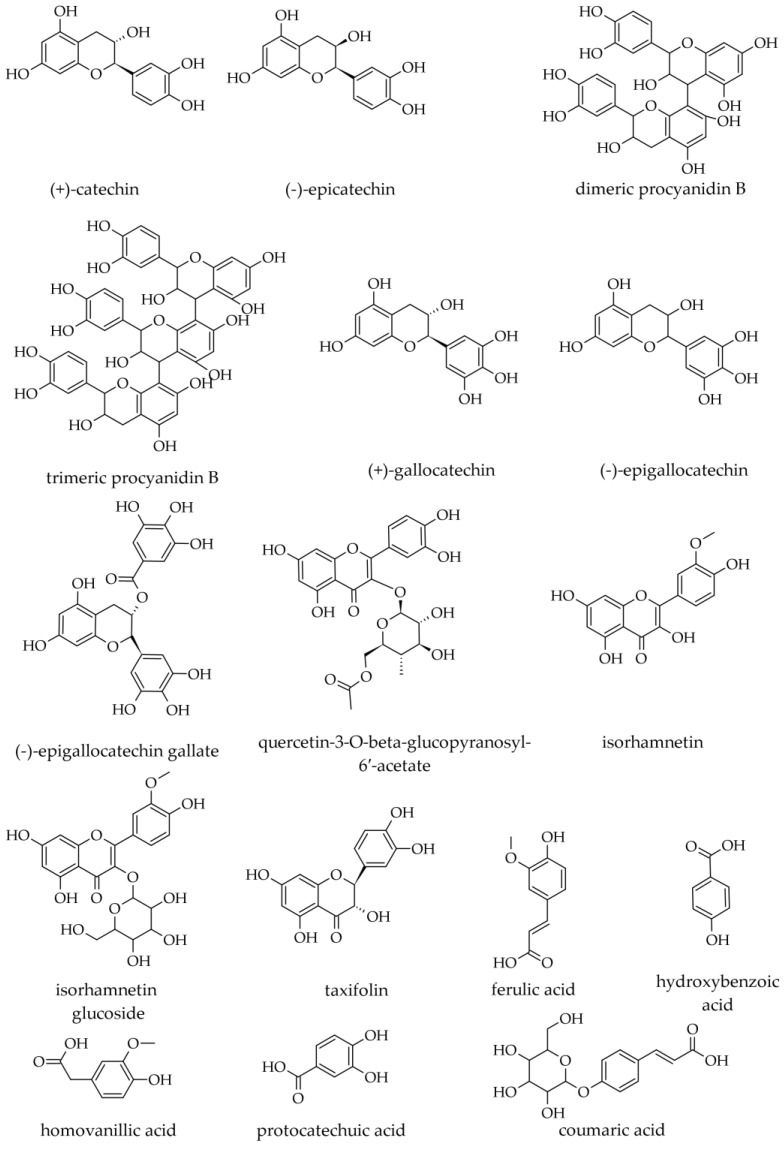
Phenolics identified in various parts of *Abies alba* Mill.

In extracts prepared with water–ethanol 50:50 (*v*/*v*), the following polyphenolic compounds have been identified: gallocatechin; epigallocatechin; gallocatechin dimer; gallocatechin gallate; (+)-catechin; quercetin glycoside (SiC), quercetin-3-O-beta-glucopyranosyl-6′-acetate, isorhamnetin, and isorhamnetin glucoside (Figure 14). Although stilbene derivatives have been reported in *Picea* spp., none have been identified in *A. alba* [54].

In water extracts prepared from trunk (stem) or branch bark, the most abundant phenolics were catechin, epicatechin, taxifolin, ferulic acid, and to a lesser extent, homovanillic and coumaric acid [18]. Catechin and epicatechin seem the most abundant and are found at similar levels in hydrophilic extracts. For catechin, bark from the trunk seems to be slightly richer than bark from branches (0.76 mg/g vs. 0.68–0.72 mg/g, d.w.), whereas, for epicatechin, this is not the case (0.78 mg/g vs. 0.66–0.81 mg/g, d.w.). On the contrary, taxifolin seems to be slightly less abundant in the bark from the trunk than in the bark from branches (0.29 mg/g vs. 0.34–0.38 mg/g, d.w.) [18].

Unlike other Pinaceae species, prodelphinidins are the dominant units in condensed tannins (having a polymerization degree ≤ 9; generally, the polymerization degree is lower for Gymnosperm tannins than for Angiosperm tannins), whereas procyanidins are less represented (their ratio is about 4:1). Procyanidins are found mostly in a cis-configuration (epicatechin, epigallocatechin), whereas prodelphinidins are in a *trans*-configuration (catechin, gallocatechin) [6,53,54].

Coumaric acid is found in slightly higher amounts in the tree bark than in branch bark (0.10 mg/g vs. 0.08–0.09 mg/g, d.w.). Ferulic acid is present at similar levels in the bark, irrespective of its origin (trunk or branches—0.03 mg/g vs. 0.02–0.03 mg/g, d.w.). Hydroxybenzoic acid seems absent from the stem bark, whereas, in the branch bark, it may be found in low amounts (0.01 mg/g, d.w.). Homovanillic acid seems to be absent in trunk bark, as well as in the bark from the branch zones close to the stem, whereas in the bark from portions farther away from the trunk, it is present in low levels (0.025 mg/g, d.w.) [18].

Abigenol^®^ (a commercial extract prepared from bark) was reported to contain the following phenolic acids: gallic acid (0.25%); homovanillic acid; protocatechuic acid (0.77%); *p*-hydroxybenzoic acid (0.10%); vanillic acid (0.11%); and *p*-coumaric acid (0.37%) [4]. In Abigenol^®^, three flavonoid compounds were detected: catechin; epicatechin; and catechin tetramethyl ether [4]. One dose of Abigenol^®^/AlbiPhenol^®^ (150 mg) contains 9.2 ± 0.1 mg of catechins. In vitro simulation studies based on digestion indicate that about 50% of the extract catechins are released from the matrix in the digestion process, whereas about 43% would be non-bioaccessible and eliminated through feces. Experiments with in vitro data using Caco-2 cells indicated that, despite the good bioaccessibility of catechins, the bioavailable fraction was below the detection limit of the HPLC method (2.7 µg/mL) [55].

#### 3.3.2. Wood Phenolic Compounds (Including Flavonoids)

In a methanolic extract from branch wood, the following phenolic compounds were identified by LC-DAD–ESI-MS/MS: gallocatechin; dimeric procyanidin B (at least four different dimers); trimeric procyanidin B; catechin; and epicatechin [18,50]. In various wood samples (sapwood, heartwood, branch wood, living or dead knots), Vek et al. (2021) reported epicatechin either as not detected or in concentrations of 0.1 mg/g. The same authors reported homovanillic acid as being absent from sapwood and varying between 0.3 and 2.3 mg/g in various wood samples, including knots. Coumaric acid was not detected in sapwood, but among various wood samples, it had the highest concentrations in living or dead knots (0.5 mg/g) [22]. In wood, ferulic acid was reported in amounts varying (depending on the type of wood) between 0.0 and 0.2 mg/g [22].

Willför et al. (2004) did not find taxifolin in stemwood and knotwood samples [21], but Vek et al. (2021) reported that it was present in concentrations of up to 0.9 mg/kg in knot wood and branch wood samples. Small amounts of quercetin were reported by the same authors in *A. alba* wood samples, in concentrations varying between 0.1 and 1.4 mg/kg, with the highest level being found in dead knots [22].

The mean content of 28.7 mg/g (gallic acid equivalents, GAE) of phenolic compounds was estimated in a sample of six branches (mostly wood, but bark included) collected in Slovenia, with multiple segments from each branch taken at different distances from the trunk (nine segments for each branch, at a distance of about 10 cm from each other). A continuous fall in phenolic contents was reported for the segment found at increasing distances from the trunk, the phenolic content being about 61% lower at 80 cm from the trunk than at 0 cm from the trunk [13].

### 3.4. Other Compounds

#### 3.4.1. Lipids and Other Lipophilic Compounds

Among four species of conifers (*Pinus sylvestris* L., *A. alba*, *Picea abies* (L.) H.Karst.), and *Larix decidua* Mill.), the bark of *A. alba* had the second lowest contents in lipids (25.73 mg/g sample, on a dry basis) and the second highest contents in fatty acids (155.70 mg/g lipid). The latter are represented by behenic (41.78%), lignoceric (21.09%), arachidic (18.02%), tricosylic (4.52%), stearic (3.49%), heptadecenoic (3.55%), undecanoic (2.20%), and *cis*-penta-decenoic (2.07%) acids [7]. Extraction of bark with n-hexane gave a yield of 4.12 mL/100 g of fresh sample. In this n-hexane extract, nine compounds were identified, representing 99.98% of its contents (in decreasing order of their abundance): di(2-ethylhexyl)-phthalate (59.83%); methylcyclopentane or cyclopentylmethane (16.63%); 13-epimanool (a diterpenoid, 6.31%); methyl cyclohexane or cyclohexylmethane (3.73%); and 3-methylhexane (3.29%) [44].

Among the four conifer species mentioned above, *A. alba* heartwood was reported to have the lowest contents in lipids (11.03 mg/g sample, on a dry basis) and the second lowest content in fatty acids (17.48 mg/g lipid). The latter consist of arachidic (37.42%), palmitic (22.02%), pentadecanoic (15.14%), margarinic (12.23%), stearic (5.83%), myristic (5.10%), and erucic (2.14%) acids [7]. Extraction of wood with n-hexane gave a yield of 1.13 mL/100 g of fresh sample. This extract is dominated by 4-hydroxy-4-methyl-2-pentanone (diacetone alcohol), which represents 73.36%. Other four compounds that, together with diacetone alcohol, represent 97% of its composition are α-cedrol (10.08%), 2,6-dimethyl-1,3,6-heptatriene (7.35%), terpinen-4-ol (4-terpinenol, S-origanol) (3.25%), and α-phellandrene (2.96%) [44].

Among seeds of four *Abies* and three *Picea* species, seeds of *A. alba* provided the richest extracted lipophilic content (41.1%), the second in decreasing order being the seeds of *A. cephalonica* (32.0%). Whereas for *Picea* sp., pinolenic acid seems to be one of the hallmark compounds in the seed lipophilic fraction; among the four *Abies* species examined, it was only present in the seeds of *A. alba*. Among tocopherols, α-tocopherol and, in smaller amounts, γ-tocopherol were reported in *A. alba* seeds [56].

Resin hydrocarbons are present in very low amounts in the seeds, their total representing less than 0.1% of all compounds. The 18-norabieta-8,11,13-triene, abieta-8,11,13-triene, abieta-7, 13-diente, and levopimaradiene were reported in *A. alba* seeds [56]. Resin aldehydes are more abundant (0.47%): levopimaral and neoabietal are the leading compounds of this group, whereas in much smaller proportions, dehydroabietal and abietal have also been reported. Resin alcohols are present in slightly higher amounts than resin aldehydes; in decreasing quantitative order, they are *cis*-abienol, neoabietol, abietol, palustrol, levopimarol, dehydroabietol, and neoabienol. Resin acids constitute about 1.8% of the lipophilic fraction. Abietic acid was the main resin acid, followed by neoabietic, levopimaric, and dehydroabietic acids; in much smaller proportions, the following resin acids were also present: monohydroxy dehydroabietic acid; monohydroxy resin acid 1; isopimaric; sandaracopimaric; and palustric acids. A small amount of resin esters (0.02%) consisted of methyl neoabietate and methyl abietate. Fatty alcohols represent about 0.05% of the lipophilic fraction and consist mainly of tetracosanol, with very small amounts of hexacosanol and nonacosan-10-ol. Sterols, detected in very low amounts (less than 0.002%), are represented by β-sitosterol and campesterol [56].

The lipophilic fraction of *A. alba* seeds contains about 0.26% free fatty acids, of which the following have been identified (in decreasing order of quantitative importance): linoleic; oleic; stearic; behenic; heptadecanoic; lignoceric; octadecadienoic; and palmitic acids. The very low amounts of fatty acid monoglycerides (<0.009%) consisted of tetracosanoic acid monoglyceride, docosanoic acid monoglyceride, and eicosanoic acid monoglyceride. Steryl esters represent 0.34% of the total lipophilic fraction, diglycerides 2.77%, and triglycerides 19.5%. Following alkaline hydrolysis, the following fatty acids were identified: myristic; pentadecanoic; palmitic; palmitoleic; 14-methyl-hexadecanoic; stearic; oleic; *cis*-vaccenic; linoleic; taxoleic; α-linolenic; linolenic; arachidic; eicosenoic (20:1(9) and 20:1(11)); eicosadienoic; sciadonic; behenic; and lignoceric [56].

#### 3.4.2. Carbohydrates

Of an extract obtained with water at 60 °C, carbohydrates represented 29.1% (16.5% free, 12.6% bound). Monosaccharides in the bark are represented by glucose (8.97 g/kg) and fructose (9.76 g/kg), with small amounts of galactose (1.06 g/kg). Sucrose was reported as the main oligosaccharide in *A. alba* bark (8.08 g/kg), whereas raffinose and stachyose are available in much lower amounts (about 1 g/kg d.w.). Bound carbohydrates (polysaccharides) contained mainly glucose (8.84 g/kg), galactose (2.06 g/kg), arabinose (1.56 g/kg), galacturonic acid (0.69 g/kg), and mannose (0.42 g/kg). With their higher content in glucose and lower content in arabinose, bound carbohydrates from *A. alba* differed substantially from other Pinaceae species analyzed [57].

Extracts prepared with 100% ethanol from knotwood contain 8.91% saccharides, 15.56% cyclitols, as well as small amounts of disaccharides and C6 monosaccharides [23]. Living knot extracts are richer in saccharides and glycitols than those derived from dead knots [22]. The levels of D-pinitol were observed to be higher in the knots located at the upper parts of the trees, while they were lower in the knots positioned at the lower parts of the tree [23].

#### 3.4.3. Inorganic Compounds

In silver fir needles, a median nitrogen content of 13.5 g/kg was estimated in samples from the Bohemian forest. For phosphorus, a median value of 1952 mg/kg was reported in Bohemian forest samples, whereas in Germany, Slovakia, and Poland, values of 1400 mg/kg have been estimated. The average magnesium level in needles is between 1971 and 2400 mg/kg, depending on the sample sources, while average calcium levels vary between 5432 and 15,500 mg/kg. For iron, average levels varying between 53.3 mg and 215 mg/kg were found; for manganese, 625 mg/kg; and for zinc, 30–63 mg/kg [58]. In needles, nitrogen, phosphorus, K, and Mg levels tend to decrease exponentially with age, whereas those of Zn, Ca, and Mn, on the contrary, increase exponentially with age. The iron levels tended to increase linearly with age, whereas Ni, Cd, and Pb levels had an erratic dynamic pattern [59]. A bark extract prepared with water at 60 °C contains about 3.8% inorganic compounds [57].

## 4. Ethnopharmacology

In the 18th century, various drinks (teas, brews, and beers) were prepared from the needles of conifers and used for the treatment of scurvy under the name “sapinette”. Whereas in France, “sapinette” should have been a decoction of “sapin du Nord” (i.e., *Picea abies*; in Canada, it was prepared from the buds of “Prussian fir”, which designated any of *A. alba*, *A. balsamea,* or *P. abies* [60]. It is interesting that Radulescu et al. (2013) reported that irrespective of the collection season (May, July, or October), shoots of *A. alba* contain lower amounts of ascorbic acid than shoots of *P. abies*, confirming the correctness of the French understanding of “sapinette”. Silver fir was the second best source of ascorbic acid in this study, as its vitamin C shoot contents tended to be higher than those of *Pinus nigra* J.F.Arnold, *Pseudotsuga menziesii* (Mirb.) Franco or *Larix decidua* (only shoots collected in October had a higher ascorbic acid level than *A. alba*) [61]. This indicates that the Canadian version was not so inadequate either.

In the olden days, the essential oils prepared from silver fir needles were used for healing bruises and treating common colds and coughs [8]. In France, needle fir oil is used against colds and other respiratory infections and is applied as a balsam, lotion, or in a bath, often together with other oils (such as eucalyptus) [30].

The Matthiolus herbal from 1590 mentions the use of silver fir sap (resin) for the treatment of foot gout and hip pain [62]. The resin was applied in Transylvania (the western part of Romania) in the treatment of furuncles and skin problems [63]. In Albania, Bosnia and Herzegovina, and Macedonia, the resin (probably from stems and branches) is used as a balm and locally applied on wounds or warts. On wounds, the resin is sometimes applied together with tobacco, the latter being used as a hemostatic [64]. In Italian folk medicine, the resin has been used for the treatment of headaches by smearing it on the temples [65]. The resin has also been traditionally used in the treatment of arthrosis in the form of a compress applied locally [66].

In ethnomedicine, its shoots, prepared as syrup, have been used in Transylvania for the treatment of coughs and as poultices for decayed teeth; they were also chewed for teeth cleaning. In the same region, as decoctions or infusions, the shoots have been used in the treatment of respiratory diseases, whereas as baths, they were employed in the treatment of rheumatism [63].

In Transylvania, the buds were used as a decoction against swollen cervical nodes, whereas the needles were applied as a compress against “liver pain” [63]. In Romania, buds and branches have been used in external preparation on wounds for their astringent properties [67].

The bark was used as a decoction against sore throats in Transylvania [63].

In Catalonia (Spain), syrup is prepared from immature cones by cutting and macerating them with sugar. The syrup can be obtained with or without boiling, the latter having the advantage of avoiding fermentation. It is used for its balsamic, antitussive, anticatarrhal, and bronchopulmonary decongestive properties [68].

## 5. Biological and Pharmacological Effects

### 5.1. Antioxidant Effects

#### 5.1.1. Antioxidant Effects of Essential Oils

In the DPPH test, the essential oil prepared from leaves and twigs at a 10% concentration had antioxidant effects similar to those of ascorbic acid. Its effects were lower on ABTS than on DPPH free radicals [35]. S. Garzoli et al. estimated an IC50 value in the DPPH test of 7.84 ± 1.70 µg/mL for a needle essential oil of *A. alba*, placing this essential oil second with respect to its antioxidant effectiveness after the one from *Pinus mugo* Turra but before those of *Pinus cembra* L. and *Picea abies*. It had a TEAC value of 3.01 ± 0.48 µM (Trolox equivalent/mg of oil). When assessed against the ABTS radical, the four essential oils assessed differed little among themselves; *A. alba* oil had an IC50 value of 44.23 ± 1.10 µg/mL and TEAC equivalent [37]. With respect to its constituents, limonene has been shown to have strong effects on DPPH and mild effects on ABTS; β-pinene has “moderate” effects on DPPH (21.4% at a concentration not clearly identified in the original report) and low effects on ABTS, whereas α-pinene has negligible effects on both free radicals [35,69,70]. Among 15 essential oils from different plant species tested at a concentration of 50 μL/mL for their effects against DPPH, the one from *A. alba* needles (the plant part is not mentioned in this paper, but it was confirmed to us by the correspondence author) was among the least effective in its scavenging effects, providing only 56.17% antioxidant activity (compared, for instance, with the strongest, from *Origanum vulgare*, which provided 93.00% antioxidant activity at the same concentration of 50 μL/mL) [71].

Upon evaluation using the DPPH method, it was found that the cone essential oil derived from *A. alba* exhibits more potent antioxidant effects compared to the oil extracted from the seeds. The antioxidant effects of the oils from cones and seeds of *A. koreana* were of intermediate potency, falling between those of *A. alba* cones and seeds. [33].

#### 5.1.2. Antioxidant Effects of Various Extracts

In extracts obtained from fresh and dried needles via hydrodynamic cavitation with water as a solvent, the DPPH antioxidant activity turned out to be very sensitive to the extraction temperature, with an abrupt fall beyond 47 °C. The DPPH antioxidant effects showed little correlation with the total polyphenol or total flavonoid contents. The ORAC antioxidant effects of the same extracts were also sensitive to temperature, but in a different manner, with sudden drops being reported when the temperature increased over a threshold of around 60 °C. The ORAC antioxidant effects correlated better with the total polyphenol and flavonoid concentrations [16].

The antioxidant effects of extracts prepared from branches decreased as their distance from the trunk increased. For extracts obtained from segments at 80 cm from the trunk, there was a 52% reduction in DPPH radical (12.1 ± 1.1 mg/g GAE vs. 5.8 mg GAE) and a 51% reduction in ABTS radical cation (11.7 mg/g vs. 5.7 mg/g) when compared with extracts obtained from segments in the trunk’s immediate vicinity. Branch extracts from segments 0 cm from a trunk had a 12.2 (±1.1) mg/g antioxidant effect against DPPH, and segments from 80 cm had a 5.8 mg/g (±2.8) antioxidant effect; the same extracts had an antioxidant effect against the ABTS radical cation of 11.7 (±0.8) mg/g GAE at 0 cm and 5.7 mg/g GAE at 80 cm [13].

Among hydrophilic extracts prepared from various wood samples, the one derived from sapwood had the lowest scavenging effect, whereas the extracts prepared from branch wood, living or dead knots, had similar scavenging effects, estimated against DPPH, at the 100 mg/mL concentration as about 60% lower than gallic acid and 30% lower than ascorbic acid, whereas at 500 mg/mL, the activity was about 20% inferior to the two reference substances (IC_50_ values were not computed) [22].

Stem bark and branch bark hydrophilic extracts, rich in lignans and phenolic derivatives, showed antioxidant effects inferior to those of gallic acid and ascorbic acid, particularly at concentrations lower than about 500 mg/L. At 1000 mg/L, extracts from both stem and branch bark have an effect virtually equivalent to that of the two reference substances (about 90% inhibition of DPPH); however, the two reference substances cause the same effect (90% inhibition of DPPH) starting from 300 mg/L upwards. At 100 mg/L, neither of the two extracts had a discernible effect on DPPH, whereas ascorbic acid inhibited about 50% of the DPPH, and gallic acid inhibited about 90% of the DPPH [18]. The ethyl acetate fraction of a bark extract had an IC_50_ value of 7.9 ± 0.1 μg/mL against DPPH and 1.56 ± 0.05 μg/mL against ferrous ions, the values being close to those of catechin (7.1 ± 0.05 μg/mL) and sodic EDTA (1.27 ± 0.1 μg/mL) [72]. Based on the IC_50_ values, the scavenging activity against superoxide (53.30 ± 5.91 μg/mL) and hydroxyl radicals (63.12 ± 1.78 μg/mL) seemed weaker [72].

For the SFTE extract (derived from the trunk), an IC_50_ value of 2.9 μg/mL was estimated for the scavenging activity against the hydroxyl radical. This effect was stronger against the same radical than that recorded for resveratrol (IC_50_ = 5.8 μg/mL), butylated hydroxytoluene (BHT, IC_50_ = 9.2 μg/mL), tocopherol (IC_50_ = 10.1 μg/mL), and epigallocatechin gallate (EGCG, IC_50_ = 18.3 μg/mL) [27].

In a cell-based assay, Abigenol^®^ (which is a bark extract) was claimed to have significantly higher antioxidant effects compared with a commercial *Pinus maritima* bark dry extract ((F0400/Pycnogenol^®^), almost a double effect compared with the *Pinus maritima* bark extract. An extract prepared by the same authors by direct extraction with ethyl acetate, followed by precipitation of polyphenols using heptane (“d-AABE”), had even stronger antioxidant activity [4]. No IC_50_ value was estimated for the antioxidant effect of the two extracts in this paper.

Abigenol^®^/AlbiPhenol^®^ significantly diminished the oxidative burden caused by diethylmaleate in the endothelium (HUVEC cells) by lowering both the formation of ROS and the oxidation of glutathione. Despite the limited availability of the extract at a concentration of 2.7 µg/mL, it still effectively lowered the ROS production caused by diethylmaleate. However, a relevant decrease in glutathione oxidation could only be achieved using 1000 µg/mL (i.e., the highest non-toxic concentration of the extract). The extract had no significant effect on the expression of superoxide dismutase (SOD) when interacting with the endothelium. The extract also diminished the oxidative stress caused by diethylmaleate in H9c2 cells, lowering both ROS formation (at 700 µg/mL) and SOD activity (both at 700 µg/mL and at 2.7 µg/mL) and having no effect on glutathione oxidation. At 1200 µg/mL, the extract significantly reduced ROS production and glutathione oxidation in HepG2 cells. In vitro, Abigenol^®^/AlbiPhenol^®^ at its bioavailable concentration level was shown to meaningfully stall HDL and LDL oxidation by 51.8% and 43.9%, respectively, whereas at higher concentrations, the antioxidant effect on the two lipoprotein fractions could reach 98.5% and 85.8%, respectively. In the ORAC (Oxygen Radical Absorbance Capacity) assay, the extract showed high values (expressed as Trolox equivalents), allowing its placement among the first four positions in the USDAORAC database (a printed edition from 2010) [55]. However, the importance of this finding should not be overemphasized. It is known that the ORAC database has been removed from the USDA website because of two chief reasons: wide misuse by the food and dietary supplement industries and concerns about the scientific validity and the in vivo relevance of the ORAC values because in vitro data could not be in a confident manner translated into clinical benefits demonstrable in appropriately designed clinical trials [73].

Belinal^®^ (an extract derived from silver fir branches) had an antioxidant activity against the ABTS, DPPH, and NO radicals that were inferior to EGCG, resveratrol, BHT, and ascorbic acid; its reducing power was also inferior to these reference substances. In the lipid peroxidation test, the extract was also inferior to BHT and resveratrol. Against the superoxide radical, Belinal^®^ was substantially inferior to EGCG, but its effects were better than those of resveratrol. Instead, Belinal^®^ was more active against the hydroxyl radical than resveratrol, BHT, and EGCG (ascorbic acid was not used as a reference in this test), suggesting its ability to pass through cell membranes inside the cells. In vivo, using an intracellular assay in *Saccharomyces cerevisiae*, Belinal^®^ demonstrated an antioxidant effect comparable to that of EGCG and superior to ascorbic acid, resveratrol, BHT, and tocopheryl succinate). The antioxidant effect increased in a non-significant manner with increasing concentrations in the in vivo conditions, whereas for the reference substances used, a decrease was observed with increasing concentrations or even a pro-oxidant effect [52].

The intracellular production of ROS by the C2C12 cells in the presence of the Belinal^®^ extract was markedly lower in a high glucose environment in comparison to a *Castanea sativa* Mill. wood extract, and the antioxidant effect was stronger than that of Pycnogenol. A decrease in ROS production was also observed in a low glucose environment, but in this case, the effect was stronger for the lower extract concentration [74].

In a simulated digestion study (in the absence of gut microbiota), Belinal^®^ showed relatively good gastric stability for the first two hours and good intestinal stability for additional two hours. However, the extract potency decreased by approximately 30% and 20% during gastric and intestinal digestion, respectively, as determined by a DPPH-based method [52].

### 5.2. Antimicrobial Activity

#### 5.2.1. Antimicrobial Activity of Essential Oils

Essential oils from various plant species have been investigated for their potential antimicrobial effects as early as 1924 [75]. In interpreting the results discussed below, one should bear in mind that in the literature, it has been estimated that a crude extract, in order to be considered to have promising activity, must have a MIC of less than 100 μg/mL and a pure compound must have a MIC of less than 16 μg/mL [76].

It has been stated that generally, essential oils tend to be more active on yeasts than on bacteria, but in a study assessing the antimicrobial effects of essential oils from nine species and sub-species taxons belonging to the *Abies* genus (twigs with foliage), the essential oil of *A. alba* was devoid of any activity against yeasts; the same essential oil was also among the least active against bacteria [75]. In an in vitro disk diffusion test, the essential oils of *A. alba* leaves, *Melaleuca armillaris* subsp. *armillaris* Sm. leaves, and *M. quinquenervia* (Cav.) S.T.Blake had all rather modest activity against three *Penicillium* species; however, the activity of the essential oil from *A. alba* leaves seemed lowest. In situ, on several foods, at the highest concentration tested (500 µL/L), the three essential oils had similar effects against *Penicillium citrinum*; the essential oil of *A. alba* leaves had an intermediate activity (among the three oils) against *P. expansum*; and the lowest activity against *P. crustosum*. Anyway, the 500 µL/L is quite high [40].

Against *Penicillium verrucosum*, the essential oil prepared from cones was more active than the essential oil prepared from needles (MIC 6.25 μL/mL vs. 9.38 μL/mL). However, both activities were modest even when compared with the oregano essential oil (*Origanum vulgare* L., 1.17 μL/mL) or pure thymol (125 μg/mL) [77].

Salamon et al. (2019) tested eleven essential oils from different plant species, including *A. alba* (plant part not stated), using a disk-diffusion method. They found that the essential oil had a weak activity or no activity at all against bacteria, but it had the second strongest activity against *Candida albicans* (ATCC 885-653); this effect was, however, was less than half of the one reported for the essential oil of *Thymus vulgaris* L. Similar results were seen when the same essential oils were tested against clinical isolates, with the difference that, in this case, the one derived from *A. alba* had the third strongest activity against *Candida albicans*, being only slightly inferior to the effect observed for the essential oil obtained from *Pinus sylvestris* L. Although the plant parts were not stated in this paper, the authors mentioned the following compounds in the essential oil composition: limonene (24.0 ± 2.0%); bornyl acetate (18.0 ± 1.0%); α-pinene (15.0 ± 1.0%); and borneol (2.2 ± 0.2%) [78].

Bağci and Diğrak [75] used a disk-diffusion method; for many bacterial species, the *A. alba* oil (twigs and foliage) was fully inactive or caused inhibition areas of 9 mm or less (only at 6.0–9.0 μg/disk concentrations; at lower concentrations it was devoid of any activity) [75]. The results are broadly consistent with a disk diffusion study performed on essential oil derived from twigs and foliage from Korean specimens. No antibacterial effects were detected on five bacterial species, whereas on *S. aures*, 25 μL of essential oil demonstrated about 70% of the inhibitory effects of gentamicin (25 μg) [35]. However, it should be taken into account that 25 μL of essential oil corresponds to a weight of about 20 mg, not 25 μg, i.e., implies a concentration about 800 times higher (assuming a density of the essential oil around 0.8 g/mL), but also a mixture with at least 30–40 compounds—many only in traces or small amounts). Mitić et al. (2022) [39] evaluated the essential oil prepared from the leaves of three *Abies* genera against 17 microbial species, and in all of them, the oil from *A. alba* failed to show any antimicrobial effect up to very high concentrations (20 mg/mL).

Garzoli et al. (2021) estimated MIC and MBC values for an essential oil derived from needles against *Escherichia coli*, *Pseudomonas fluorescens*, and *Kocuria marina* to 51.28 mg/mL, a huge value compared with the cut-off of interest of 100 μg/mL. For *Acinetobacter bohemicus* and *Bacillus cereus*, the estimated MIC values were slightly lower, 12.82 mg/mL and 25.64 mg/mL, but they are still very high compared with the threshold value of interest. The same authors also assessed the antibacterial effects of the essential oil in the vapor phase with a disk diffusion method and found no effect against *E. coli* and *P. fluorescens*, whereas its activity against *A. bohemicus*, *K. marina* and *B. cereus* were evaluated as stronger than in the liquid phase [37]. Furthermore, if the objective is, for example, to provide a pleasant scent in a room while reducing microbial burden, applying the oil in the vapor phase might be more feasible.

In a study evaluating the antimicrobial effects of 15 essential oils from sundry plant species, the essential oil of *A. alba* needles was found to be the most active against *Clostridium butyricum*, *Clostridium intestinale*, and *Clostridium ramosum*. However, MIC values for this essential oil against those species were 0.25, 1.70, and 0.25 μL/mL [71]. This corresponds to about 20 μg/mL, 1360 μg/mL, and 20 μg/mL, which are still above the 16 μg/mL cut-off value for considering them as having promising activity. Compared with the effects reported for *A. alba* essential oils against other bacterial species, these effects against *Clostridium* seem substantially stronger. It is interesting that the chemical composition of this essential oil was rather different from the one reported for the majority of oils obtained from this species: bornyl acetate (30%); camphene (18%); α-pinene (3%); borneol (1.5%); and α-terpinene (1.2%) (the remainder of the compounds are not stated in the reference source) [71].

Șandru (2015) evaluated 12 essential oils from different plant species for their effect on *E. coli* using a disk diffusion method [79]. The essential oil of *A. alba* (plant parts not specified, no chemical composition provided) was among the most active, with inhibition zones of 17.3–18.1 mm (depending on the time of measurement). However, the author used high amounts of essential oil, 100 μL/disk, equivalent to about 80 mg/disk.

Limonene, which is one of the major compounds of *A. alba* essential oils, has been described in that paper that evaluated multiple natural terpene ingredients to have an MBC of 6000 μg/g (6 mg/g) or higher against several bacterial species [80], despite the fact that experimental evidence showed that limonene was able to disrupt the lipidic profile of microbial cytoplasmic membranes [81]. In another study, MIC values of 2–27 mg/mL were reported for limonene, which confirms a rather low antimicrobial effect [82].

In an in vitro study, MIC values for (+)-α-pinene ranged between 117 and 3125 μg/mL for three fungal species, whereas the MIC for the same isomer against MRSA was 4.1 mg/mL; the (−)-α-pinene enantiomer was reported to be completely devoid of antimicrobial activity. MIC values for (+)-β-pinene were between 187 and 780 μg/mL for the same fungal species, and the MIC for the same isomer against MRSA was 6.2 mg/mL; the (−)-β-pinene enantiomer was also fully devoid of antimicrobial activity [83]. In another evaluation, for Gram (+) bacteria, the MIC values of α-pinene (enantiomer not stated/checked) varied between 0.75 and 1.29% (*v*/*v*), whereas for β-pinene (enantiomer not stated/checked), they varied between 1.16 and >2.0% (*v*/*v*); for Gram(−) bacteria, the MIC values for α-pinene varied between 1.05 and 1.59% (*v*/*v*), while for β-pinene they varied between 1.80 and >2.0% (*v*/*v*) [84].

Camphene also has a rather modest antibacterial activity, with various studies reporting MICs for representative Gram (+) and Gram (−) pathogens larger than 120 µg/mL [85]. As for bornyl acetate, MIC values higher than 1 mg/mL were reported against *S. aureus*, *S. epidermidis*, and a set of Gram-negative bacilli [86]. α-Terpineol and terpinen-4-ol have MICs lower than α-pinene against *Staphylococcus aureus*, *S. epidermidis*, and *Propionobacterium acnes*, but they are only present in small amounts in this essential oil. In that same paper [87], the authors found a stronger effect for α-pinene than for α-terpineol and terpinen-4-ol in TLC-bioautography; this, though, is inconsistent not only with the MIC values of the same study but likewise, with the minimal bactericidal concentration (MBC) determined by Cosentino et al. (1999) for α-pinene, which was over 900 μg/mL [88]. Borneol is also reported to have some antibacterial effects (inferior to chloramphenicol) [89] and is also present in low amounts in *A. alba* essential oils. *p*-Cymene, γ-terpinene, and linalool are not only present in low amounts in the essential oils of *A. alba* products but their MBC values have also been estimated at over 900 μg/mL for all bacterial species on which they were tested [88].

The essential oils prepared from seeds and cone scales also have relatively low antimicrobial effects compared with thymol as a positive control. The essential oils from *A. alba* seeds and cone scales had less inhibitory effects than those from *A. koreana*. Therefore, it is unlikely that the antimicrobial effects of the essential oils from seeds or cones would be of real clinical interest. Similarly, essential oils prepared from leaves or twigs have only modest antimicrobial activity, whereas those from *A. koreana* have been described as stronger. The minimal inhibitory concentrations (MICs) against different Gram (+) and Gram (−) bacteria varied between 10.0 and 28.5 μL/mL for essential oils obtained from *A. koreana* seeds or cones and between 16.5 and 33.0 μL/mL for those prepared from *A. alba* products [33].

Lanzerstorfer et al. (2019) reported that dispensing a mixture of essential oils from *Citrus limon* (L.) Osbeck (plant part not stated, but from the available commercial info of the product, it is derived from the pericarp) and *A. alba* (part not stated, but from the available commercial info of the product, it is derived from needles and branches) with ultrasonic vaporizers (type VASE) (2–3 mg m^–3^), a mean decline in bacteria concentration of about 40% was observed for two hours following air dispersion. In the case of fungal species, a mean reduction of 30–60% was reported, depending on the ward where the oils were applied [41].

To conclude, an impressive amount of research has consistently reported high MIC values for *A. alba* essential oils (in comparison with the indicative cut-offs mentioned above) and high MIC values reported for the leading compounds of those essential oils. Thus, the low antimicrobial activity reported up to now for *A. alba* essential oils is in line with their chemical composition and with common knowledge about the antimicrobial activity of their main ingredients.

#### 5.2.2. Antimicrobial Activity of Various *A. alba* Extracts

An 80% methanol needle extract was tested against three phytopathogenic fungi, and it either was fully devoid of any activity (*Fusarium oxysporum*, *Phytophthora cambivora*) or had a low activity level (*Alternaria alternata*) [90].

Hydrophilic extracts rich in lignans and various phenolic compounds were also devoid of or had minimal antifungal activity on *Trametes versicolor*, *Schizophyllum commune*, *Gloeophyllum trabeum*, and *Penicillium expansum* [18]. Knot wood and branch wood extracts from *A. alba* had a very weak inhibitory effect on *Trametes versicolor, Schizophyllum commune*, and *Gloeophyllum trabeum*, with 5% solutions causing an average fungal inhibition of about 20% and 1% solutions causing no inhibition at all. The situation was similar for two mold species tested, *Penicillium expansum* and *Fusarium solani*, with about 25% inhibition observed for 5% of solutions and about 10% inhibition for 1% of solutions [22]. These findings were in strong agreement with those reported by Välimaa et al. (2007), who investigated a large number of knotwood or bark hydrophilic extracts derived from 30 tree species, with both soft (i.e., gymnosperms) or hardwood (angiosperms). In the preliminary screening, they found that the silver fir knotwood extract was completely inactive against most bacterial and yeast species tested; the only positive effect was recorded against *B. cereus*, but it was very weak (11% inhibition). This lack of effect was also confirmed in more in-depth assessments when the same extract also proved to be devoid of almost any effect (for most species tested, the average inhibition was 0% or close to 0%; for *Candida albicans,* a 15% inhibition was reported, but this dwarfed in comparison with *Pinus* species extracts, for which inhibition was over 80%) [91].

### 5.3. Cytotoxicity and Toxicity

#### 5.3.1. Cytotoxic Activity of *A. alba* Essential Oils

The essential oil derived from *A. alba* leaves and twigs had no effect on human fibroblast cells (CCD-986 SK line) survival for 24 h at concentrations of 1% or lower, but at 5%, it quickly and significantly reduced the cells’ ability to survive, going down to 34% after 6 h and 6.3% after 24 h [35].

Essential oils prepared from the leaves of three *Abies* species (*A. alba*, *A. cephalonica*, and *A. borisii-regis*) were evaluated for their toxicity against nauplii of *Artemia salina,* and all were found to manifest relatively strong toxicity (LC less than 100 mg/L), but among the three, the essential oil from *A. alba* was estimated as having the lowest toxicity. The authors speculated that the lower toxicity of this essential oil compared with the other two would be related to its high contents of camphene and limonene, which are considered less toxic among the mono- and sesquiterpenes of the three essential oils. The same three essential oils also demonstrate some toxicity against *Drosophila melanogaster* larvae, with the one from *A. alba* also having the lowest toxicity [39].

Essential oils from seeds and cones from *A. alba* (as those from *A. koreana*) were weakly active against MCF-7 (human breast cancer) and MDA-MB-231 (very aggressive, invasive, and poorly differentiated breast cancer) cell lines. The IC_50_ values, around 100 μg/mL, were very close to the IC_50_ values estimated in normal human fibroblasts. This indicates very limited selectivity and a lack of potential for the use of these essential oils against cancer. The authors hypothesize that this low effect is related to the fact that in both cells, the dominant compound was the (−) limonene isomer, whereas based on the literature data, (+) limonene would have interesting antitumor effects without causing organ dysfunction. However, the authors concluded that at concentrations in the 1–50 μg/mL range, the oils “were rather safe”. The two essential oils were less toxic than thymol for normal human cells [33].

#### 5.3.2. Cytotoxic Activity of Various *A. alba* Extracts

Abigenol^®^/AlbiPhenol^®^ appeared to have no significant effect on the intestinal epithelium’s health or permeability. The maximum concentration at which Abigenol^®^/AlbiPhenol^®^ was non-toxic was 1000 µg/mL for HUVEC cells, 700 µg/mL for H9c2, and 1200 µg/mL for HepG2 cells [55].

Belinal^®^ triggered a significant drop in surviving mesenchymal stem/stromal cells (MSCs) (about 20% of cells remaining viable); a slightly more pronounced effect (in the same sense) was observed for Pycnogenol, resveratrol, and quercetin. However, in that experiment, even for control, the viability of 50–60% was observed; therefore, the authors chose the 375 µg/mL concentration for further experiments (which was the lowest concentration tested) [25]. Low concentrations (5 or 10 μg/mL) of Belinal^®^ have no impact on the murine myoblast cell line C2C12 viability [74].

Knotwood extracts derived from *A. alba* were virtually innocuous on Hepa-1 cells [91].

### 5.4. Influence on Chondrogenesis

In vitro, compared with control and pycnogenol, resveratrol, and quercetin, Belinal^®^ promoted chondrogenesis in human bone-derived mesenchymal stem/stromal cells (hMSCs) from osteoarthritis patients. Such an effect, though, was not observed for MSCs obtained from healthy donors (post-mortem), with the authors speculating that this difference could be due to the lower chondrogenic potential of hMSC derived from osteoarthritis patients compared with those from healthy donors [25].

### 5.5. Prebiotic Effects

Stojanov et al. (2021) reported on the effects of a commercial wood extract (Belinal^®^), which reportedly contains a variety of lignan molecules, as well as phenols and carbohydrates, on ten distinct *Lactobacillus* species, several of which were human isolates. They found that the extract acts as a prebiotic for several of these species: *L. paracasei, L. rhamnosus*, and *L. acidophilus* (of intestinal origin), *L. gasseri* and *L. crispatus* (of vaginal origin), and *L. bulgaricus* (a species employed in the food industry). The ingredients of the extract that act as prebiotics have not been identified, although the authors speculated that they could be oligosaccharides or other carbohydrates [26].

### 5.6. Antidiabetic Effects

Starting from the antidiabetic effects reported for Pycnogenol, attributed to the inhibition of α-amylase and α-glucosidase, Lunder et al. tested Belinal^®^ as well as an extract prepared from *A. alba* bark to assess their effects on these same enzymes, as well as on dipeptidyl peptidase 4 (DPP4). This study used high concentrations of extracts based on the reasonable assumption that following oral administration, such high concentrations are achievable. The two *A. alba* extracts exhibited what the authors described as “great in vitro inhibitory potential” for all three enzymes. For α-glucosidase, Belinal^®^ had the lowest IC_50_ estimated value (1.4 mg/mL), whereas the bark extract had an IC_50_ of 2.1 mg/mL, the exact same estimate (2.1 mg/mL) reported for Pycnogenol. These IC_50_ values are lower than the one estimated for Farmatan^®^, an industrial extract derived from *Castanea sativa* wood [74].

In the case of α-amylase, the strongest inhibitory effect was observed for Pygnogenol (IC_50_ 1.5 mg/mL), followed by the *A. alba* bark extract (IC_50_ 3.3 mg/mL). Belinal^®^’s effect was weaker (11.8 mg/mL); only the *C. sativa* wood extract had a higher IC_50_ value (38.2 mg/mL) [74].

The *A. alba* bark extract had the strongest inhibitory effect on DPP4 (IC50 3.4 mg/mL) among the evaluated extracts, followed by Pycnogenol (IC_50_ 4.8 mg/mL), Belinal^®^ (IC_50_ 5.2 mg/mL), and Farmatan (IC_50_ 9.1 mg/mL) [74].

Although the enzymatic inhibitory effects of these extracts were described as “effective”, their value is still relatively high, considering that, for instance, for the fucoidan extracted from algal species *Ascophyllum nodosum,* IC_50_ was lower than 0.05 mg/mL [92], but because relatively high concentrations can be achieved in the gut, these findings could be of clinical relevance.

The authors evaluated nine lignans (lariciresinol, matairesinol, nortrachelogenin, pinoresinol, secoisolariciresinol diglucoside, 7-hydroxymatairesinol, isolariciresinol, pinoresinol diglucoside, and secolariciresinol) found in *A. alba* extracts. They found that pinoresinol diglucoside and isolariciresinol, at a 1 mg/mL concentration, cause notable inhibition of DPP4 (almost 50% inhibition); all others were less active on DPP4 and completely inactive or only weakly active on α-glucosidase and α-amylase. At the same concentration (1 mg/mL), Belinal^®^ caused about 40% inhibition of α-glucosidase, 15% inhibition of α-amylase, and 32% inhibition of DPP4. However, lignans seem not to be the only compounds responsible for the antidiabetic effects, as the same authors have shown that at least three natural phenolic acids (gallic, protocatechuic, and *p*-hydroxybenzoic) are moderate inhibitors of α-amylase, whereas *p*-coumaric acid is an inhibitor of α-glucosidase [74].

Belinal^®^’s efficacy in controlling glycemia was evaluated in a small clinical trial (*n* = 31) on healthy volunteers with a normal BMI and a normal response to the oral glucose tolerance test [93]. This study was double-blind, randomized, and employed a cross-over design implying a baseline visit plus four test visits with a washout period of one week. On each of the four occasions, the subjects consumed 100 g of white bread and received one of the trial products: a placebo; 200 mg of Belinal^®^; 200 mg of chestnut extract; or 50 mg of acarbose. After 90 min, glycemia values were significantly high for the placebo and the chestnut extract but not for acarbose or Belinal^®^. After 120 min, the concentrations among the four groups did not differ significantly from the initial fasting values. The authors concluded that Belinal^®^ was only slightly inferior to acarbose but superior to the placebo and chestnut wood extract. The mean post-prandial insulin concentration was about 30% lower in the acarbose group (*p* = 0.048), 25% lower in the Belinal^®^ group (*p* = 0.010), and only 4% lower in the chestnut wood group (*p* > 0.05; value not stated in the source publication). The glycemic index for the standard meal consumed with Belinal^®^ was 65%, and consumed with acarbose was 57%; both were statistically significantly different from the standard meal consumed with placebo (100%) but not statistically different among themselves (*p* = 0.27). The glycemic index of the standard meal consumed with chestnut wood extract was 87% and did not differ significantly from the standard meal taken with placebo [93]. The results are encouraging, but considering the limitations of this study (small sample size bias, lack of transparency on treatment assignment, and a population consisting of healthy volunteers and not diabetic patients), there is a need for confirmation in clinical trials properly designed and performed.

### 5.7. Hepatic Steatosis

In an in vitro model of hepatic steatosis, using the HepG2 liver cell line and oleic acid as a trigger for fat accumulation, the Abigenol^®^/AlbiPhenol^®^ extract markedly decreased lipid accrual in the hepatic cells induced by oleic acid at 0.5 mM, but not when oleic acid was used at the 1 mM concentration. The extract triggered a 68.8% reduction in cholesterol in HepG2 hepatocytes when used at a concentration of 1200 µg/mL but not at the low bioavailable concentration of 2.7 µg/mL. Limited evidence suggested that this effect may be partially explained by a slight increase in the biosynthesis of bile acids as a result of cholesterol reduction [55].

### 5.8. Cardiovascular Effects

Ex vivo, in a model of ischemia-reperfusion injury, the phenol-rich SFTE extract was reported to have a substantial cardioprotective effect on isolated rat hearts. *p*-Coumaric and protocatechuic acids, hypothesized by the authors to be active ingredients of the extract, had an effect inferior to the one of the extract. LDH release rates (related to ischemia severity and myocardial damage) were mitigated by SFTE in a concentration-dependent manner (the effect being more pronounced at 100 µg/L than at 10 µg/L), whereas *p*-coumaric and protocatechuic acids were devoid of such effects. SFTE also caused a concentration-dependent rise in coronary flow comparable to the one seen with *p*-coumaric acid (but not with protocatechuic acid); this indicates a vasodilatory effect, speculated by the authors to be partly related to the interference with the NO synthase signaling pathway and partly related to the antioxidant effects of the extract. The fact that L-NNA, an eNOS inhibitor, suppressed this effect confirms the NO-synthase pathway mechanism. Although SFTE had no significant impact on the heart rate, the authors stated that it “appeared to restore the pre-ischemic heart rate values”. A significant reduction by 80% of the entire duration of arrhythmias in the reperfusion period was claimed in the same ex vivo study for SFTE. This was an effect size larger than the one observed for the two natural acids (40% reduction by *p*-coumaric acid and 45% reduction by protocatechuic acid) [94]. All in all, this set of experiments indicates a cardio-protective effect for the SFTE extract, but non-clinical and clinical confirmation remains necessary.

The SFTE extract (silver fir trunk extract), rich in polyphenols, also showed protective effects against the negative effects on arterial function and the changes in artery morphology induced by an atherogenic diet in guinea pigs [27,94]. The thoracic aorta rings of animals pre-contracted with phenylephrine relaxed under acetylcholine. However, in the case of animals subjected to an atherogenic diet, the extent of this relaxation was drastically diminished, whereas SFTE strongly improved the relaxation effect. It is hypothesized that this vasorelaxant effect is at least partly related to a NO-related mechanism but certainly not by a direct action on smooth muscle cells. The inside of the guinea pig’s abdominal aortas showed a significant increase in atherosclerotic plaques compared with the negative controls. However, including SFTE in the diet responsible for atherosclerosis significantly reduced atherosclerosis occurrence (by about 80%) [27].

At 700 µg/mL, the Abigenol^®^/AlbiPhenol^®^ extract significantly inhibited angiotensin-converting enzyme (ACE) activity in H9c2 cells but not in HUVEC cells at 1000 µg/mL. As the effect size for ACE inhibition in H9c2 cells was modest (an inhibition of about 10–15%) [55], the practical significance of this finding appears unclear at best in the absence of additional data.

### 5.9. Anti-Psoriatic Effects

An ointment based on silver fir bark extract (ABI) 2%, rich in polyphenols, was evaluated in a small (*n* = 61, out of which 56 completed the trial), randomized, double-blind, placebo-controlled, right–left clinical trial in patients with psoriasis. The ointment was applied for 12 weeks or until the treated area was clean. The extract significantly and substantially inhibited IL-1β, but otherwise, it had no significant effect on IL-6, IL-8, IL-10, IL-12p70, and TNF-α levels. The fall in IL-1β levels is attributed to the polyphenol fraction, as a similar effect was also observed for a similar *Pinus pinaster* bark extract. A 15% difference in improving symptoms was reported by the authors in favor of the extract against placebo, but it was not statistically significant (and this could be related to the small sample size used in the trial, but even if assuming that the effect was significant, its size is relatively small) [95].

### 5.10. Antitumour Effects

Karkabounas et al. (2000) reported the effects of an aqueous extract derived from a mixture of *A. alba* and *Viscum album se abies*, i.e., mistletoe living as a parasite on silver fir. On L-1210 cells (murine lymphocytic leukemia), an IC_50_ value of 49.6 ± 1.4 mg/L was estimated. In a (male) Wistar rat model of carcinoma induced by benzo(α)pyrene (BaP), the authors claimed a significant extension of life and a decline in the tumor growth rate for the extract in animals treated with BaP compared with the control group, a modest effect of inhibiting tumor induction (by 16.6%), and a life extension and necrotic effects induced on malignant cells, when the extract was administered in animals with pre-formed tumors induced by BaP [96].

### 5.11. Pharmacokinetics

In vitro data suggest that gastrointestinal digestion of Belinal^®^ does not have a significant impact on lignan molecules [25] in the sense that they are not substantially degraded. No in vivo pharmacokinetic data are available for *A. alba* extracts, neither in animals nor humans.

## 6. Conclusions

In the last three decades, an impressive amount of research has been dedicated to the *A. alba* species. These are mainly phytochemical studies; however, there have also been a number of studies on the biological or pharmacological effects of volatile oils or extracts obtained from different parts of the plant. The variability of essential oils (whether they come from leaves, oleoresin from branches, or other parts of the plant) is impressive, even among specimens collected from the same geographical area. The period of the collection also appears to substantially influence the chemical composition of essential oils. For essential oils prepared from needles or twigs and branches, limonene, β-pinene, α-pinene, camphene, β-phellandrene, and bornyl acetate are the leading compounds, although their wide variations seem to correspond to multiple chemotypes. Supercritical fluid extraction from the same plant parts results in essential oils poorer in monoterpenes and much richer in sesquiterpenes (27.3–30.0%) than steam distillation (where sesquiterpenes represent at most 3–4%). Essential oils obtained from cortical oleoresin are also richer in sesquiterpenes than needle essential oils (13.5–30.8% depending on several variables, including season of collection), although the major monoterpenes are about the same (limonene, β-pinene, α-pinene, camphene, bornyl acetate). The investigations conducted thus far have revealed that α-pinene is the predominant compound in the cone essential oil, along with other monoterpenes such as limonene, β-pinene, and verbenone. From the limited research available up to date, the seed essential oil is dominated by limonene (70.1–82.9%) and α-pinene (6.3–11.5%). Both bark and wood are rich in lignans and phenolic compounds. Matairesinol is apparently the dominant lignan in bark, and secoisolariciresinol and lariciresinol are the dominant ones in wood samples.

Essential oils and sundry extracts (from different parts of the plant) seem to have non-negligible antioxidant effects. Instead, both various essential oils and extracts from different parts of *A. alba* seem to either be devoid of antibacterial effects or to have only very modest such effects. Both essential oils and various extracts are relatively safe in their effects on multiple cell lines, with little to no cytotoxicity. Encouraging results have been reported in several in vitro or non-clinical experiments performed with a variety of extracts with respect to their prebiotic effects, their positive effects on chondrogenesis and hepatic steatosis, as well as their antidiabetic, cardiovascular, and antitumor effects. Their anti-psoriatic effects seem too limited to be of real clinical interest.

It is expected that the future will bring to line new data with respect to both the phytochemistry and biological and pharmacological activity of the species. Correlating phytochemistry with the genetic makeup of the specimens analyzed would improve our understanding of the wide variability observed, particularly with respect to essential oils; discovering that epigenetic aspects are as equally important, though, should not take by surprise the scientific community. Most pharmacological research has been focused to date on more or less characterized extracts, but identifying key active compounds and their mechanisms of action in the case of positive results should remain a priority for future research. Furthermore, it is expected that new therapeutic areas will be explored in the future, using a variety of extracts and pure compounds derived from *A. alba*.

## Figures and Tables

**Table 1:** Variation of monoterpene hydrocarbon contents in Albanian samples of needle essential oil collected in different seasons [19].

Monoterpene	Proportion in Winter Samples (%)	Proportion in Summer Samples (%)
β-pinene	24.7–31.6	14.5–18.4
camphene	10.8–17.2	3.0–5.4
limonene	9.6–17.5	6.5–10.2
α-pinene	9.5–11.7	8.4–15.2
santene	2.1–3.2	0.3–1.0
tricyclene	1.3–1.8	0.3–0.7
myrcene	1.0–1.2	0.6–0.8
α-terpinolene	0.6–0.8	0.8–1.7
α-phellandrene	0.1–0.2	0.1–0.2
Z-ß-ocimene	traces—0.2	traces

**Table 2:** Variation of oxygenated monoterpene contents in Albanian samples of needle essential oil collected in different seasons [19].

Oxygenated Monoterpene	Proportion in Winter Samples (%)	Proportion in Summer Samples (%)
Bornyl acetate	5.68–17.6%	1.6–2.4%
α-Terpineol	1.21–2.0%	1.21–2.0%
Borneol	0.4–1.7%	2.3–5.5%
α-Terpinyl acetate	0.35–1.4%	0.2–0.4%
Linalool acetate	0.1–1.0%	Traces
Linalool	0.1–0.92%	traces—0.2%
Camphene hydrate	traces—0.2%	0.2–0.4%
*p*-Menth-1-en-9-ol-acetate	traces—0.2%	0.2–0.7%
α-Campholenal	traces—0.1%	0.2–0.5%
Camphor	traces—0.1%	0.1–0.2%
*cis*-Pinocamphone	traces—0.1%	0.1–0.3%
Terpin-4-ol	traces—0.1%	traces—0.4%
Endo-fenchol	Traces	0.2–0.4%
Trans-pinocarveol	Traces	0.4–1.0%

**Table 3:** Variation of sesquiterpene hydrocarbon contents in Albanian samples of needle essential oil collected in different seasons [19].

Sesquiterpene Hydrocarbon	Proportion in Winter Samples (%)	Proportion in Summer Samples (%)
E-caryophyllene	0.6–1.2%	7.2–12.8%
Neryl acetate	absent/traces	0.2–0.6%
α-himachalene	traces—0.2%	traces—0.28%
α-humulene	0.3–0.6%	4.7–7.2%
9-*epi*-E-caryophyllene (2-*epi*-(E)-β-caryophyllene)	traces	traces—0.4%
y-gurjunene	traces	traces—0.4%
y-muurolene	traces	0.5–0.9%
Germacrene D	0.1–0.3%	0.1–0.3%
β-selinene	traces—0.2%	0.6–1.0%
*cis*-β-guaiene	traces—0.4%	0.3–1.2%
α-selinene	0.0–0.2%	0.5–1.0%
β-himachalene	0.2–0.4%	0.7–1.2%
Germacrene A	traces—0.1%	traces—0.4%
y-cadinene	traces—0.1%	0.1–1.2%
δ-cadinene	0.3–0.5%	2.0–3.5%
Cadina-1,4-diene	0.1%	0.2–0.3%
α-cadinene	traces	0.2–0.3%
α-calacorene	traces—0.3%	0.1–0.3%

**Table 4:** Variation of oxygenated sesquiterpene contents in Albanian samples of needle essential oil collected in different seasons [19].

Compound	Winter Samples (% Composition)	Summer Samples (% Composition)
E-nerolidol	traces—0.4%	0.1–0.4%
10-*epi*-γ-eudesmol	1.4–5.4%	0.7–3.9%
*epi*-α-cadinol	traces—0.6%	0.2–0.3%
cubenol	0.2–0.5%	traces—0.1%
himachalol	0.3–0.8%	traces—0.2%
α-cadinol	0.3–0.42%	2.7–4.2%
E,E-farnesol	traces—0.3%	0.3–1.0%

**Table 5:** Variation of monoterpene hydrocarbon contents in Albanian samples of bark essential oil collected in different seasons [19].

Monoterpene Hydrocarbon	Proportion in Winter Samples (%)	Proportion in Summer Samples (%)
α-pinene	12.2–37.2	23.1–47.1
camphene	0.3–0.9	0.4–0.9
β-pinene	14.9–36.6	18.3–30.0
myrcene	1.4–1.6	1.4–2.6
limonene	6.9–35.3	5.5–41.4
α-terpinolene	traces—0.1	traces

**Table 6:** Variation of sesquiterpene hydrocarbon contents in Albanian samples of bark essential oil collected in different seasons [19].

Compound	Proportion in Winter Samples (%)	Proportion in Summer Samples (%)
α-longipinene	traces—0.3%	traces—0.1%
neryl acetate	traces—0.2%	traces—0.1%
E-caryophyllene	3.4–8.9%	3.2–3.9%
α-himachalene	traces—0.3%	traces
α-humulene	1.6–3.9	1.6–1.9
y-muurolene	0.3–0.6%	traces—0.1%
germacrene D	5.9–15.2%	6.9–12.1%
β-*cis*-guaijene	traces—0.7%	traces—0.1%
α-selinene	traces—0.3%	traces—0.2%
β-himachalene	traces—0.2%	traces—0.1%
γ-cadinene	0.2–0.6%	0.1–0.3%
δ-cadinene	0.6–1.1%	traces—0.3%
α-cadinene	0.3–0.6%	0.2–0.4%

## Data Availability

Data sharing is not applicable.
